# Advancing Hydrocephalus Management: Pathogenesis Insights, Therapeutic Innovations, and Emerging Challenges

**DOI:** 10.14336/AD.2024.1434

**Published:** 2024-12-29

**Authors:** Xiuyun Liu, Hui Zhi, Marek Czosnyka, Chiara Robba, Zofia Czosnyka, Jennifer Lee Summers, Huijie Yu, Xiaoguang Tong, Guoyi Gao, Gelei Xiao, Kai Yu, Yan Xing, Renling Mao, Shaoya Yin, Yangong Chao, Hongliang Li, Ke Pu, Keke Feng, Meijun Pang, Dong Ming

**Affiliations:** ^1^State Key Laboratory of Advanced Medical Materials and Devices, Medical School, Tianjin University, Tianjin, China.; ^2^Haihe Laboratory of Brain-Computer Interaction and Human-Machine Integration, Tianjin, China.; ^3^School of Pharmaceutical Science and Technology, Tianjin University, Tianjin, China.; ^4^Department of Clinical Neurosciences, Addenbrooke's Hospital, University of Cambridge, Cambridge, CB2 0QQ, UK.; ^5^San Martino Policlinico Hospital, IRCCS for Oncology and Neuroscience, Genoa, Italy.; ^6^Department of Anesthesiology and Critical Care Medicine, Johns Hopkins University, Baltimore 21287, USA.; ^7^Department of Neurosurgery, Tianjin Medical University General Hospital, Tianjin, China.; ^8^Department of Neurosurgery, Tianjin Huanhu Hospital, Tianjin, China.; ^9^Department of Neurosurgery, Beijing Tiantan Hospital, Capital Medical University, Beijing, China.; ^10^Medical School, Beijing Normal University, Beijing, China.; ^11^Department of Neurology, Aviation General Hospital, Beijing, China.; ^12^Department of Neurosurgery, Huadong Hospital, Fudan University, Shanghai, China.; ^13^Department of Critical Care Medicine, The First Hospital of Tsing Hua University, Beijing, China.

**Keywords:** hydrocephalus, nervous system diseases, cerebrospinal fluid, pathology, genetic techniques, therapeutic innovations

## Abstract

Hydrocephalus is a prevalent neurological disorder, particularly impactful in older adults, characterized by high incidence and numerous complications that impose a significant burden on healthcare systems. This review aims to provide a comprehensive description of hydrocephalus pathogenesis, focusing on cellular and molecular insights derived from animal models. We also present the latest advances in hydrocephalus research and highlight potential therapeutic targets. Lastly, the review advocates the integration of findings from both animal and human studies to achieve better outcomes and examines the potential of emerging technologies. We wish to raise public attention about this disease in an aging society. Current animal models for hydrocephalus involve acquired hydrocephalus models and genetic/congenital hydrocephalus models. Studies from animals have shown that the main mechanisms of models can be broadly classified into nine types. A variety of drug-targeted therapy methods and non-surgical treatment methods have been used in clinical practice. But current treatment approaches primarily focus on symptomatic relief and intracranial pressure control rather than addressing the underlying pathological mechanisms. We call for the development of more accurate and representative animal models to achieve better outcomes and examine the potential of emerging technologies, such as artificial intelligence and neuroimaging. In summary, this review synthesizes recent findings in hydrocephalus research, identifies promising therapeutic targets and interventions, and critically evaluates the limitations of current research paradigms, aiming to align preclinical studies with clinical endpoints. Continued studies and multidisciplinary collaboration are essential to develop effective interventions and facilitate new treatments into bedside.

## 1. Introduction

Hydrocephalus is a prevalent neurological disorder characterized by the dilated ventricles, combined with a series of clinical symptoms, including progressive gait disturbances, cognitive impairment, and urinary incontinence [1-3]. The incidence rate of hydrocephalus is as high as 175 per 100,000 among older adults (≥65 years) [4, 5]. Disruptions or blockage of the cerebrospinal fluid (CSF) production, circulation, or absorption can result in hydrocephalus, leading to intracranial hypertension, decline of motor and visual abilities, and even life-threatening conditions if left untreated. Hydrocephalus can be categorized into the congenital (primary, and/or genetic) or the acquired (secondary) hydrocephalus, with the former group mainly caused by genetic factors and the acquired hydrocephalus primarily associated with trauma (post-hemorrhagic hydrocephalus, PHH), infections (post-infectious hydrocephalus, PIH), etc. [3]. Conventional treatments for hydrocephalus include CSF shunting and third ventriculostomy, both of which are associated with high complication rates and failure rates reaching 40-50% [6].

**Figure 1.**
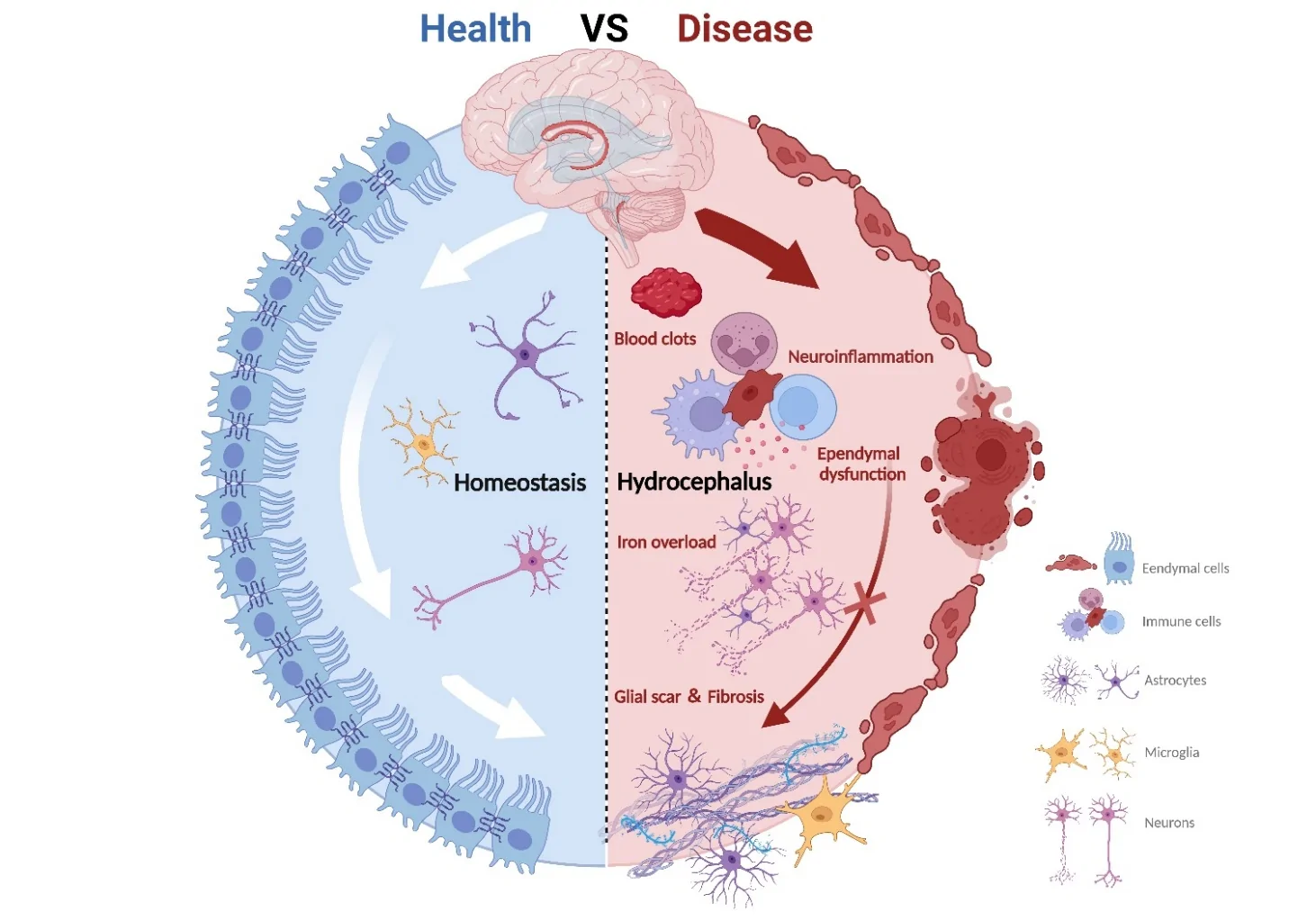
**The pathophysiology of hydrocephalus**. The schematic diagram on the left shows the normal circulation of CSF. Any disruption in the processes of CSF circulation, such as production, movement, or absorption, can lead to the occurrence of hydrocephalus, as depicted on the right. Neuroinflammation, blood clots, iron overload, fibrosis, and ependymal dysfunction are recognized as significant mechanisms of hydrocephalus.

Researchers have identified several mechanisms of hydrocephalus including neuroinflammation, iron overload, and ependymal dysfunction, as summarized in Fig. 1. Karimy et al reviewed the evidence that neuroinflammation plays a pivotal role in pathogenesis of hydrocephalus by promoting CSF hypersecretion and fibrosis [3]. Similarly, Strahle et al. pointed out that the penetration of blood components from vessels to CSF may cause the overload of irons resulting in abnormal expression of aquaporins (AQPs), blood-brain barrier (BBB) disruption, oxidative stress, and fibrosis [7]. Furthermore, dysfunction of the ependymal cells, such as ependymal denudation, impaired ciliary movement, and disorders of gliocyte proliferation, is another main reason of hydrocephalus [8].

However, most of the above findings are based on animal studies, with only less than 5% genes being identified in humans [9, 10]. Compared with other common brain disorders, a comprehensive understanding of pathogenesis of hydrocephalus has the potential to facilitate new treatments of this prevalent disease. We sought to provide a comprehensive review of hydrocephalus, from the perspective of CSF dynamics, animal models, pathogenesis, and the therapeutic targets. The objectives of this review are to (1) provide an overview of animal models in hydrocephalus research, covering genetic modifications, induced models, and the relevance of large animal models to human anatomy and physiology; (2) synthesize recent advances in our understanding of hydrocephalus pathogenesis; (3) highlight potential therapeutic targets for hydrocephalus treatment; (4) discuss research gaps, challenges, and future needs. We wish to raise public attention about this disease in an aging society and inspire new ideas for hydrocephalus treatment.

## 2. Pathophysiology of Hydrocephalus

### 2.1 Mechanisms of CSF dynamics

Early theories of CSF circulation viewed CSF as a passive, relatively static fluid, without a clear understanding of its dynamic flow or continuous production and absorption. In the 20th century, the classical CSF circulation model proposed that CSF was secreted by the choroid plexus, flowed through the ventricles, meninges, and the subarachnoid space surrounding the spinal cord, and was eventually absorbed into the venous sinuses via the arachnoid granulations [11]. It was thought to drain through perineural spaces to the nasal mucosa and peripheral lymphatic vessels in the neck. However, the classical model struggled to explain how large molecules were efficiently cleared from the brain efficiently [12].

With advancements in modern imaging technologies, a more refined concept of CSF circulation has emerged, emphasizing the role of the glymphatic system (the glial-lymphatic system) in CSF dynamics [13]. In 2017, the existence of lymphatic vessels in the human brain was confirmed through high-resolution clinical magnetic resonance imaging (MRI), which further expanded our understanding of the glymphatic system [14-16]. The glymphatic (glial-lymphatic) pathway consists of three anatomically distinct regions: the periarterial space, the perivenous space, and the brain parenchyma [13]. Within this system, CSF in the subarachnoid space exchanges dynamically with interstitial fluid (ISF) between neurons and glial cells, particularly within the perivascular space (also known as the Virchow-Robin space). This exchange is influenced by various factors, including osmotic and hydrostatic pressure gradients between the blood, perivascular space, and brain parenchyma, as well as arterial pulsation, respiration, consciousness, and head position [11, 16-20]. AQP4, which is located on the endfeet of astrocytes and particularly polarized around the periarterial space, facilitates CSF movement into the brain parenchyma, where it interacts with ISF [13, 16-20]. The fluid then drains back through the venous perivascular and perineuronal spaces into the subarachnoid space and the lymphatic system [21]. Ultimately, metabolic waste products and toxic proteins in the CSF are cleared through meningeal lymphatic vessels to the cervical lymph nodes and the peripheral lymphatic system [13, 22]. This bidirectional flow is regulated by circadian rhythms, encompassing both CSF secretion and absorption [23].

### 2.1.1 Production of cerebrospinal fluid

Over 80% of CSF is produced by the choroid plexus, with the remainder generated by the brain parenchymal system, which is regulated by nerve fibers (such as the sympathetic and cholinergic nerves) and hormonal factors [24, 25]. The choroid plexus is an epithelio-endothelial convolute, composed of highly vascularized stroma with connective tissue and fenestrated capillaries, along with epithelial cells. It is located in the lateral, third, and fourth ventricles. The lack of tight junctions in endothelial cells of the choroid plexus capillaries facilitates the production of CSF. Current research indicates that the main driving forces for CSF secretion are the hydrostatic pressure gradient and osmolarity between the capillaries, choroid plexus epithelial cells, and ventricles [26, 27]. According to Starling's forces, net fluid movement across the plasma membrane of capillaries is governed by the interplay of hydrostatic and oncotic forces, which facilitates the passive filtration of blood plasma into the choroidal interstitial compartment (i.e., the space between the choroidal capillaries and choroid epithelial cells) [24]. Sodium transport is a key factor, with Na^+^, HCO^3-^, and Cl^-^ ions moving into the choroid plexus epithelial cells across the basolateral membrane [25]. Ion transport is regulated by Na^+^-K^+^-ATPase, as well as K^+^ co-transporters such as KCC3, KCC4, and KCNE2, which facilitate K^+^ transport [11, 28]. Transporters located on the apical membrane of the choroid plexus epithelial cells actively transport Na^+^, Cl^-^, K^+^, and HCO^3-^ into the ventricular space, creating an osmotic gradient that draws water molecules from the blood into epithelial cells and eventually into the CSF. Carbonic anhydrase, which catalyzes the formation of HCO^3-^ from water and carbon dioxide, also promotes this process. The Na^+^-K^+^-2Cl^-^ cotransporter NKCC1 is responsible for over half of CSF production, while AQP1 accounts for 20-25% of CSF formation [29-31].Additionally, the glucose transporter-1 (GLUT1) facilitates transcellular water transport, while paracellular pathways through tight junctions also contribute to water movement [32, 33]..

Approximately 10-20% of CSF is generated through CSF-ISF exchange in the brain parenchyma, a process that is more efficient during sleep [34, 35]. The capillary-astrocyte complex is thought to be the primary site for CSF-ISF production and exchange, a process likely facilitated by the absence of tight junctions between pial cells or ependymal cells [11, 34, 35].

Under normal physiological conditions, the choroid plexus regulates CSF production through active transport mechanisms and the expression of aquaporins. However, when choroid plexus cells undergo hyperplasia or pathological changes, CSF production may significantly increase, surpassing the brain's capacity for absorption and drainage. This imbalance leads to the accumulation of CSF within the ventricular system, resulting in elevated intracranial pressure and the development of hydrocephalus. The overproduction of CSF can be attributed to several biochemical and molecular mechanisms. For example, upregulation of aquaporins plays a critical role in modulating CSF secretion. Additionally, enhanced activity of ion transporters, such as the NKCC and Na^+^-K^+^- ATPase, can contribute to increased CSF production [3, 36, 37].

### 2.1.2 Circulation of cerebrospinal fluid

The understanding of CSF circulation has been continuously refined, with new insights emerging. CSF movement is now recognized as a complex, pulsatile flow influenced by ciliary beating, cardiac pulsations, and body movements, as visualized through magnetic resonance flow imaging [38-40]. The current model of CSF circulation suggests that CSF is initially produced in the lateral ventricles and then flows through the foramen of Monro into the third ventricle. After pooling in the third ventricle, the CSF passes through the cerebral aqueduct (also known as the Sylvian aqueduct) into the fourth ventricle. From there, it enters the subarachnoid space via the foramina of Luschka and Magendie, where it surrounds the brain and spinal cord [11].

CSF circulation is a complex process driven by multiple mechanical and physiological factors. One of the primary drivers of this flow is the coordinated movement of ependymal cilia lining the ventricular system, which beat in a synchronized manner, facilitating directional CSF flow. Additionally, pulsations associated with the respiratory cycle and fluctuations in vasomotor tone contribute to CSF dynamics. In rodent models, changes in body posture also have been shown to significantly impact CSF flow [13, 14, 16, 18]. These findings highlight the dynamic nature of CSF circulation, emphasizing that it is not merely a passive process but is actively driven by both intrinsic and extrinsic forces.

When a physical obstruction impedes CSF flow, non-communication or obstructive hydrocephalus may ensue. This condition is frequently associated with congenital anomalies, such as aqueductal stenosis, where the narrow cerebral aqueduct between the third and fourth ventricles becomes blocked. This obstruction prevents normal CSF flow from the lateral and third ventricles to the subarachnoid space. The resulting accumulation of CSF upstream of the blockage leads to ventricular dilation and increased intracranial pressure. The mechanical pressure exerted by this buildup can compress the periventricular white matter, disrupt neural conduction, and cause cognitive and motor impairments [3, 5, 24, 41-43].

### 2.1.3 Absorption of cerebrospinal fluid

The pathways for CSF absorption include arachnoid villi drainage, lymphatic drainage, and glymphatic drainage [11]. Arachnoid granulations (also referred to as Pacchionian granulations) are structures located between the arachnoid and dura mater on the surface of the brain. These granulations facilitate the absorption of CSF into the venous sinuses via a hydrostatic pressure gradient between the CSF and the dural venous blood, eventually draining into the internal jugular system [44]. While arachnoid villi drainage has long been supported by anatomical evidence and was traditionally regarded as the primary route for CSF absorption, recent research suggests that lymphatic drainage may play a more significant role [44, 45]. The proportion of CSF drained via the lymphatic system is estimated to range from 5% to 50% [46-48]. CSF outflow through the lymphatic drainage system involves perineural sheaths around cranial and spinal nerves as well as meningeal lymphatic vessels [49, 50]. Specifically, CSF travels along the perineural sheaths surrounding cranial and spinal nerves, entering the lymphatic drainage system [24]. And meningeal lymphatic vessels, which are situated around the dural sinuses, middle meningeal artery, and cribriform plate, connect the intracranial and extracranial lymphatic systems and play a crucial role in the clearance of extracellular tau proteins and immune cells [51]. CSF entering the meningeal lymphatic vessels from the subarachnoid space, along with CSF cleared via the glymphatic pathway, is eventually drained into the venous system or into the deep cervical lymph nodes [52]. In humans, one of the primary exit points for CSF through the perineural pathways is the cribriform plate region of the nasal cavity. CSF can penetrate the nasal mucosa along the olfactory nerve through the foramina of the cribriform plate, ultimately draining into the lymphatic vessels of the nasal mucosa and subsequently into the cervical lymph nodes [53, 54]. Due to the technical limitations of monitoring CSF drainage, different drainage routes face difficulties in quantification. There is still controversy over the proportion of drainage volume of different routes.

Reduced CSF absorption is typically linked to communicating hydrocephalus. Inflammatory responses, such as those seen in infections like meningitis or following events like intraventricular hemorrhage that deposit blood breakdown products in the subarachnoid space, can impair the function of arachnoid granulations (Fig. 1). When the reabsorptive capacity is compromised, CSF accumulates within the ventricular system, leading to its dilation and the subsequent development of hydrocephalus [55].

### 2.2 Classification system for hydrocephalus

Hydrocephalus is defined as "an active distension of the ventricular system resulting from inadequate passage of CSF from its point of production within the cerebral ventricles to its point of absorption into the systemic circulation" [56]. The pathophysiology of hydrocephalus fundamentally arises from an imbalance among the production, circulation, and absorption of CSF. Rather than being a single pathological entity, hydrocephalus represents a pathophysiological condition characterized by disturbed CSF dynamics, with a wide range of clinical manifestations and disease trajectories [57]. This multifactorial nature implies that the disorder encompasses various complex classifications, including classifications based on onset, CSF dynamics, intracranial pressure levels, imaging features, and genetic background [8, 57]. While binary classification systems based on clinical features and CSF dynamics are widely used, no system yet fully captures the comprehensive characteristics of hydrocephalus. Historically, hydrocephalus has been categorized into non-communicating and communicating forms, based on the bulk flow theory. These refer to hydrocephalus caused by obstructed CSF flow within the ventricular system and hydrocephalus resulting from impaired CSF absorption, respectively, a classification still commonly used today [24]. In the case of the kaolin-induced hydrocephalus model, injecting kaolin into the subarachnoid space can lead to localized physical obstruction of CSF pathways. However, the inflammatory response triggered by the kaolin may extend throughout the meningeal system, impeding CSF reabsorption and demonstrating features of both communicating and non-communicating hydrocephalus [58]. Similar complexities are observed in chronic hydrocephalus patients [59, 60]. To address the multifactorial nature of hydrocephalus and advancements in imaging technologies, the "Multi-Categorical Hydrocephalus Classification" (Mc HC) was proposed, which incorporates CSF circulation dynamics to more precisely characterize hydrocephalus subtypes [57]. This multi-dimensional and comprehensive hydrocephalus classification system can more accurately reflect the individual state of hydrocephalus patients and assist doctors in formulating personalized treatment plans, which have high clinical practicability. We look forward to the further improvement and application of the hydrocephalus classification system.

### 2.3 Overview of animal models in hydrocephalus research

Animal models are indispensable tools for studying the pathophysiological mechanisms of hydrocephalus and developing therapeutic strategies. Their significance lies in elucidating the pathophysiology of hydrocephalus and evaluating novel therapeutic interventions. This is largely attributable to the high degree of anatomical and physiological similarity between animal models and humans. Moreover, the controlled experimental conditions of animal models make them ideal for pathological investigations at the anatomical, cellular, and molecular levels. These models provide critical targets for genetic screening, facilitate the prediction of disease progression, and enable the evaluation of the efficacy and safety of novel therapies, thereby offering valuable evidence for the clinical diagnosis and treatment of hydrocephalus in humans.

Rodents, particularly mice and rats, are the most commonly used species in hydrocephalus research. Their small size, short generation times, and well-established genetic tools make them ideal for controlled experimentation. There are two major categories of rodent models: acquired hydrocephalus models and genetic/congenital models. Acquired hydrocephalus models of hydrocephalus in rodents are created through surgical, chemical, or physical interventions to simulate the various forms of hydrocephalus, including obstructive and communicating types. Some widely used methods include the injection of kaolin or blood into the ventricle of the animals [36, 61-63]. These models have demonstrated several pathological changes associated with hydrocephalus, such as deformation of neural cells due to compression, disruption of BBB, which is a selective barrier formed by endothelial cells of brain capillaries, supported by astrocytic end-feet and pericytes, and demyelination of white matter [64-66]. Furthermore, brain tissue atrophy observed in rat models aligns with findings from human MRI studies, providing critical pathological insights for the imaging-based diagnosis of hydrocephalus [67-69]. In contrast, genetic or congenital hydrocephalus models involve either naturally occurring mutations or targeted genetic modifications introduced via CRISPR-Cas9 or traditional transgenic methods. These models are particularly valuable for investigating the pathogenesis and genetic underpinnings of congenital hydrocephalus. For instance, congenital hydrocephalus animal models generated using gene-editing techniques (e.g., Daple-deficient mice and Dvl TKO^hGFAP-Cre^ mutant mice) provide an essential experimental platform for elucidating the genetic basis of human congenital hydrocephalus [70, 71].

Larger animal models, such as dogs, pigs, and non-human primates, are used to bridge the gap between rodent studies and clinical applications. These models are more anatomically and physiologically similar to humans, providing a better understanding of hydrocephalus in the context of human-like CSF dynamics and brain architecture. For example, beagle pups were widely used in germinal matrix hemorrhage (GMH) models in the 1980s [72]. The germinal matrix layer of beagle pups has immature blood vessels, which are similar to those of humans. Moreover, they are of great application value in the research fields of behaviors such as movement and sensation. In addition, due to the relatively large size of large animals, it is easier to measure and control their physiological parameters. In the early research on hydrocephalus, dogs or pigs were mostly chosen as experimental subjects in order to test the therapeutic effect of plasminogen activators [73-75].

Therefore, animal models serve as a critical bridge between basic research and clinical practice. The safety and efficacy data obtained from animal experiments provide a reliable foundation for subsequent human trials. It is essential for advancing our understanding of hydrocephalus, promoting pathophysiological research, and evaluating and optimizing novel therapeutic strategies.

## 3. Animal model in Hydrocephalus Research

The physiological and pathological features of an ideal animal model should be closely similar to those observed in humans. This involves pathological and functional validation, including histological analysis and imaging to confirm hallmark features of hydrocephalus, such as ventricular enlargement, brain tissue atrophy, white matter lesions, and altered CSF biochemical components. Behavioral and cognitive assessments (such as memory tests in rodent models) are performed to ensure that the model exhibits neurological deficits similar to those of human patients. Some models assess pre- and post-treatment responses to evaluate the efficacy of therapeutic interventions. For example, the role of neuroprotective agents in reducing ventricular dilation and improving neurological outcomes in the model is a crucial step in validating the model's utility [76].

This review classifies animal models of hydrocephalus into acquired and genetic/congenital types based on modeling methods and pathogenic mechanisms. Understanding their achievements in replicating or simulating the potential molecular, cellular, and anatomical changes of clinical hydrocephalus will help us better seek preventive strategies and improve patient prognosis.

## 3.1 Insights gained from acquired hydrocephalus models

### 3.1.1 Post-hemorrhagic hydrocephalus models

Primary intraventricular hemorrhage (PIVH) is a subtype of intraventricular hemorrhage (IVH), signifying non-traumatic intracranial bleeding occurring within the ventricular system and the adjoining ependymal membrane, lacking hemorrhagic parenchymal components [77-82]. Hydrocephalus occurs in over half of PIVH cases, making it a prevalent complication, with around one-third requiring ventricular drainage [77]. We have summarized most of the PIVH models in the eTable in the Supplement. The most commonly employed technique in establishing PIVH-induced hydrocephalus models is the direct injection of autologous blood into the ventricles. Initially, large animals such as dogs or pigs were commonly selected due to easy manipulation and monitoring. Subsequently, PIVH-Induced hydrocephalus has also been successfully modeled in adult and neonatal rodents [83, 84]. It is apparent that blood injection is more prone to induce hydrocephalus than artificial CSF (aCSF) injection, and bilateral injections are more predisposed to hydrocephalus than unilateral injections [78]. In recent years, similar models have emerged that utilize specific blood components, such as iron and lysophosphatidic acid (LPA) [85, 86]. Injection of lysed red blood cells and FeCl_3_ both results in acute hydrocephalus [87]. In embryonic mice, severe hydrocephalus occurs after LPA injection [79]. LPA disrupts ventricular integrity and causes third ventricle occlusion by directly affecting the migration and fate of neural progenitor cells in embryonic mice [79]. In neonatal mice injected with LPA solution, ventriculomegaly is observed in all cases, resulting from ventricular surface denudation caused by ependymal cell damage and ciliary dysfunction, with no evidence of physical obstruction [88]. In adult rats, LPA acts directly on TRPV4 channels, leading to overactivation of NKCC1, promoting excessive CSF secretion, and consequently causing hydrocephalus [86]. It can be seen that in the same type of models at different developmental stages, their intrinsic pathogenic mechanisms also vary.

GMH refers to traumatic or spontaneous rupture of immature capillaries within the subependymal brain tissue of premature infants, also known as periventricular hemorrhage [89, 90]. It is the most common cause of acquired hydrocephalus in infants and a leading cause of mortality in preterm and very low birth weight (VLBW) newborns [24, 91]. The GMH model induced hydrocephalus in newborns by injecting materials, such as collagenase, which is able to disrupt the extracellular matrix, thus inducing vascular rupture of the germinal matrix, also known as ganglionic eminence [92, 93] (eTable in the Supplement). The advantages of the collagenase model lie in its induction of slow spontaneous bleeding. Although the acute mass effect is not apparent, it avoids the confusion of brain injuries and/or infarction caused by rapidly injecting a large volume of blood into the ventricles, leading to increased intracranial pressure (ICP) [94, 95]. From a practical application perspective, the collagenase model has high repeatability, low labor intensity, and bleeding volume directly correlates with enzyme injection. This makes it suitable for comparing treatment targets and testing bleeding complications. Importantly, this model induces spontaneous bleeding, accompanied by an inflammatory response for about a week, but it does not cause major neurofunctional defects, making it more akin to the clinical conditions of human preterm infants [93]. Injection of autologous whole blood into the periventricular regions, such as the GM and striatum, has been proven to induce mild enlargement of the ipsilateral ventricle in neonatal mice [96, 97]. Blood components like iron and hemoglobin have also been used in the construction of GMH-associated hydrocephalus models. Guo et al. observed a significant increase in iron-positive cells and heme oxygenase-1 (HO-1) protein levels on the first day after modeling [98]. Subsequently, injection of hemoglobin and iron into the right ventricle of postnatal day 7 (P7) rats was shown to successfully induce hydrocephalus, with ventricular volume positively correlated with injection concentration. However, injection of protoporphyrin IX, the iron-deficient immediate heme precursor, and aCSF did not result in significant ventricular enlargement. The lack of ventricular enlargement with protoporphyrin IX injection may be due to its iron-chelating activity and inhibition of HO-1 [98, 99]. Some GMH models are established by intraperitoneal injection of glycerol in young rabbits, which have immature germinal matrix blood vessels, similar to humans [100]. Newborn rodents are common choices for GMH-related hydrocephalus animal models, with P7 rats being used most commonly, due to the fact that it simulates the physiological conditions of human neonates [101]. The region of myelination in P7 rats is sensitive to hypoxic-ischemic stress and is similar to the corresponding region in human fetuses or preterm infants [102]. However, this fact needs further clarification.

Intracerebral hemorrhage (ICH) refers to non-traumatic intracerebral hemorrhage resulting from the rupture of cerebral blood vessels, constituting the most prevalent cause of IVH [103]. IVH ensuing from ICH poses a potentially higher risk of sustained hydrocephalus compared to PIVH [104]. Current post-ICH hydrocephalus models are mainly divided into two categories: the injection of autologous whole blood or collagenase into the striatum [105, 106] (eTable in the Supplement). While autologous blood injection effectively induces hydrocephalus, but it cannot replicate the clinical phenomenon of vascular rupture, even worse, it may lead to blood reflux [106]. The injection of collagenase, due to its diffusion characteristic, leads to multiple vascular ruptures and bleeding, and simulates the physiological process of spontaneous vascular bleeding and following hydrocephalus. The model has a high successful rate, but high doses of collagenase injection may be toxic to neurons. The specific differences identified in the comprehensive comparative study between injection of blood and collagenase include: (1) the blood model implies larger hematomas and faster hematoma resolution; (2) the collagenase model implies more severe blood-brain barrier disruption, neuronal loss, cortical reduction, and white matter injury; (3) tissue loss volume in the blood model remains almost unchanged from 1 to 6 weeks, while tissue loss persists for 4 weeks in the collagenase model; (4) functional deficits recover faster and more completely in the blood model, making the collagenase model more suitable for assessing the effectiveness and side effects of clinical treatment methods [95]. Considering the significant differences and limitations among these models, these two methods can be used in combination and comparison.

Subarachnoid hemorrhage (SAH) is a stroke subtype primarily caused by intracranial aneurysms, characterized by the typical development of cerebral vasospasm [107]. Intracranial aneurysm refers to an abnormal dilation of blood vessels, manifested by localized damage to the cerebral artery wall, loss of internal elastic lamina, and changes or malformation in the middle layer [108]. Studies indicate an incidence of hydrocephalus after SAH ranging from 15% to 37% [109]. The mechanisms underlying acute and chronic hydrocephalus after SAH may share similarities and intertwine, with acute hydrocephalus typically considered non-communicating and chronic hydrocephalus regarded as communicating [11, 110]. In comparison to models above, there has been relatively limited investigation into animal models simulating hydrocephalus associated with SAH, which can be categorized into two types: injection models and endovascular perforation models [111, 112] (eTable in the Supplement). For experiments aimed at observing the acute consequences of SAH, a single injection is typically chosen. In contrast, to study the pathological processes of delayed injury, multiple long-term injections are required. Common injection sites in injection models include the cerebellomedullary cistern, the chiasmatic and basal cisterns, the prechiasmatic cistern, the bilateral major cerebral arteries, the high cervical internal carotid artery, and extracranial arteries. Injected substances include autologous blood, autologous blood clots, or blood components. The endovascular perforation model involves making a distal cut on the left external carotid artery, inserting a nylon monofilament suture into the stump of the external carotid artery, and advancing it through the bifurcation of the common carotid artery into the internal carotid artery to create a perforation [60]. Results showed that within 24 hours after vascular perforation, 44% of rats developed acute hydrocephalus, exacerbating both cerebral hemorrhage and ventricular injury [107, 113]. This technique holds potential for application in larger animals in future studies.

**Table 1:** Animal models of post-infectious hydrocephalus (PIH).

Age	Species/Gender (F/M)	Injection of agent	Methods	Type of hydrocephalus	Features	Conclusion	Ref.
**Neonatal/** **P1-4**	Syrian hamster/-	Non-neuroadapted mumps virus	Intracerebral inoculation	C 2w-3m	A virus induced model of aqueductal stenosis hydrocephalus described for the first time	Initial infection was predominantly in ependymal cells lining the ventricles	[114]
**Neonatal/** **P2-4**	Hamster/-	0.02 ml of an inoculum containing M. pneumoniae	Intracerebral inoculation	SA 7d-14d	No mycoplasma was found in the ependyma; the differences in the brain anatomy of hamsters make it difficult to directly compare with other studies	The mechanism might be toxic product-mediated dysfunction of CSF secretion or absorption	[115]
**Neonatal/** **P3-5**	Wistar rat/-	Mouse hepatitis virus, MHV-A59 strain	Intracerebral inoculation/intranasal inoculation/ intraperitoneal inoculation	C 2w	Intracerebral inoculation in rats produced hydrocephalus more frequently	This model had no aqueduct stenosis and might be caused by viral destruction of developing neural tissue	[36]
Adult	Rat/M	10 μl of the tumor cell suspension	Intraventricular inoculation	C 28d-30d	The implantation of this model had a high success rate; It fills the gap in the research on cerebrospinal fluid metastasis of intracranial malignancies and related treatments	This model was associated with significant neurological impairment	[116]
	**C 57 black mouse/**-	0.03 ml of 106/mi suspension of human glioma cells	Parietal lobe inoculation	C 2w-4w	Communicating hydrocephalus model	There was a hypersensitive reaction	[117]
	**Wistar rat/M**	50 cysts of Taenia crassiceps	Cisterna magna inoculation	C 3m	Albendazole intervention; the detection time points and indicators in this study may not comprehensively capture the treatment effect of albendazole	The drug was ineffective with short-term treatment	[118]
	**BALB/c mouse, BALB/c nude mouse, ICR/Slc mouse/**-	A chorioallantoic membrane-grown strain (Ska) of HSV-1	Cerebral hemisphere inoculation	C 4w	Obstructive hydrocephalus model; Lack of long-term observation and functional assessment	T cell-mediated immune effects were involved in the progression of hydrocephalus	[119]

Abbreviations: F, Female; M, Male; P, Postnatal day; A, Acute hydrocephalus; SA, Subacute hydrocephalus; C, Chronic hydrocephalus; d, Day; w, Week; m, Month; CSF, Cerebrospinal fluid.

TBI is a leading cause of death among individuals aged 15 to 30 in the United States, accounting for 2.2% of the total mortality in the country [120, 121]. Early ventricular dilation in traumatic brain injury patients can progress to post traumatic hydrocephalus (PTH) [122, 123]. Brain injuries can be induced in animal models through focal impact, diffuse impact, or a combination of both, such as weight drop models, fluid-percussion injury models and controlled cortical impact models (eTable in the Supplement). The applied loads of the impact are precisely controlled and used to reflect the severity of injuries. However, a persistent challenge is balancing the necessary simplifications in replicating clinical pathological conditions with the inherent complexities of experimental models.

### 3.1.2 Post-infectious hydrocephalus models

PIH is the most common cause of pediatric hydrocephalus, yet systematic studies on its microbial spectrum and infection pathways remain lacking [124]. Viruses and parasites are frequently employed in the construction of PIH models [36] (Table 1). Cytomegalovirus (CMV), mumps virus, Toxoplasma gondii (T. gondii), enterovirus 71 (EV71), reovirus, and lymphocytic choriomeningitis are pathogens associated with acquired hydrocephalus, representing the second major etiology [114, 125-132]. These pathogens are commonly used to construct models for PIH. Parasitic infections leading to CNS diseases represent a significant public health concern. Neurocysticercosis, resulting from the infection of the pork tapeworm, is one such condition that can give rise to cysts within the brain parenchyma, CSF spaces, as well as cystic lesions in the eyes, orbits, and spinal cord [133]. Subsequent leptomeningitis, accompanied by impaired CSF absorption, mechanical obstruction, and inflammation, further contributes to the development of hydrocephalus [134, 135]. This phenomenon occurs in 16% to 51% of patients with neurocysticercosis [136]. In 2019, Hamamoto Filho, P. T., and colleagues established a rodent model of neurocysticercosis-associated hydrocephalus by intracisternal inoculation of cysts from the small tapeworm Taenia crassiceps, along with an antigenic suspension [36]. They observed that the incidence of hydrocephalus was 57% in the group treated with kaolin, 60% in the cyst group, and 9% in the antigen group. Researchers speculated that cyst-induced mechanical obstruction might be the primary mechanism underlying hydrocephalus in this model [36]. Moreover, this model also identified periventricular astrogliosis and high expression of AQP4, suggesting a potential pathway for CSF absorption under inflammatory conditions [36]. This model contributes valuable insights into understanding the mechanisms and potential therapeutic interventions for hydrocephalus associated with neurocysticercosis.

**Table 2:** Others acquired hydrocephalus models.

Age	Species/Gender (F/M)	Injection of agent	Methods	Type of hydrocephalus	Features	Conclusion	Ref.
**Neonatal/P7**	Wistar rat/M	0.04 mL of sterile kaolin suspension	Intracisternal injection	SA 8d-14d	Hyperbaric intervention; The key mechanisms have not been deeply explored	Hyperbaric oxygen therapy alleviated brain injury in rats with hydrocephalus	[137]
**Neonatal/P10-14**	Rabbit/-	Suspension of kaolin in 0.9 % saline	Intracisternal injection	SA 2w	No evidence of ependymal disruption was found	The intercellular clafts at the ependymal junction of the lateral ventricle widened and the density of cilia decreased	[138]
**Prenatal/E100-120**	Pregnant sheep/F	A silastic heparinized catheter	Fetal aqueduct obstruction	NA	ICP could be measured from a catheter	This model was able to document the relationship between ICP, histopathological changes, and gestational age	[139]
**Juvenile/P33-41**	Pig/-	1.25 mL of sterile 25% kaolin	Intracisternal injection	SA-C 11-42d	Ventriculoperitoneal shunts intervention	A reliable model of gyrencephalic animals with acquired juvenile hydrocephalus	[140]
**Neonatal/P14**	Ferret/-	Kaolin	Intracisternal injection	C 29d	Nimodipine intervention; Species differences and brain maturity may significantly affect the evaluation of the effect	Nimodipine treatment was ineffective	[141]
**Prenatal/E13**	Pregnant mother SD rat/F	8 mg 6-AN/kg body weight	Intraperitoneal injection	A-SA 1d-8d	The fourth ventricular outlet was closed	Changes in ICP correlated with CBF	[142]
**Prenatal**	pregnant mother rat/-	8 mg 6-AN/kg body weight	Intraperitoneal injection	SA 8d	Immunoreactive cells could penetrate the ependymal lining of the lateral ventricle	Rats with hydrocephalus had marked VD and thinning of the cerebral cortex and corpus callosum	[143]
Adult	Cat/-	Acrylic screw	Aqueduct stenosis	C 3w	Characteristics of CSF pulsation	The occurrence of hydrocephalus was related to the compliance of the spinal dural sac	[144]
	**Hounds/**-	0.3-0.6mL cyanoacrylic gel	Intraventricular injection	C 12-16w	There is a close link between hypoxia and angiogenesis resulting from hydrocephalus	VEGF/VEGFR-2+ might be a therapeutic target for hydrocephalus	[145]
	**Domestic felines/F**	10 mg sterile colloidal suspension of kaolin	Intracisternal injection	SA 8d	Significant improvements have been made to the feline hydrocephalus model, but the sample size is relatively small	This model reduced mortality	[146]
	**Hamsters/F**	0.03 mL of sterile kaolin suspension	Intracisternal injection	C 15d	A study of the time series of hydrocephalus-related parameters	Cisternal injection of kaolin could cause enlargement of all ventricles and cerebral aqueducts with the third ventricle enlarging on the first day after injection	[147]
	**Rabbit/M**	Silicone oil	Intracisternal injection	C 4w-8w	CSF shunts intervention; The issue of hippocampal damage required attention	CSF shunts could not treat pathological changes in chronic hydrocephalus	[148]
	**Mongrel dog/**-	Silicone oil	Subarachnoid space injection	C 2m	An ultrastructural study of the CP	CSF secretion in the CP of dogs with chronic hydrocephalus might be disturbed	[149]
	**Mongrel dog/M**	Cyanoacrylic gel glue or liquid cyanoacrylic glue	Intraventricular injection	C 4w	Without local compression or inflammation	Intrinsic brain compliance was a key factor in the experimental hydrocephalus model	[150]
	**C57BL/6J mouse/M**	Kaolin	Intracisternal injection	A 3d-5d	A new perspective on the pathogenesis of hydrocephalus was proposed	Microglia and UPRmt were activated after hydrocephalus, which might lead to apoptosis of neuronal cells	[151]
	**SD rat/M**	30μL sterile suspension of 3% kaolin	Intraventricular injection	C 2w	The therapeutic effect of MC was verified	The Wnt inhibitor sFRP-l delayed the development of hydrocephalus and alleviated reactive gliosis	[76] [152]
	**CD1 mouse/**-	Cellulose acetate film	Placed into the atrium of the aqueduct of Sylvius for 60 or 120 days	C 2m-4m	Reversible model of hydrocephalus	This model of chronic hydrocephalus showed no death or motor impairments, with non-progressive reactive glial cell proliferation	[153]
	**SD rat/Albino guinea pig/**-	β’-iminodipropionitrile	Intraperitoneal injection	SA 1w	Acetazolamide intervention; This model is innovative, but its universality remains to be determined.	The mechanism of this model induced communicative hydrocephalus is excessive secretion of CSF by the CP	[154]
	**SD rat/M**	Human recombinant VEGF-A165	Intraventricular injection	A 6d	Antiangiogenic drugs might be effective in patients with hydrocephalus	VEGF infusion could cause VD and ependymal denudation	[155]
	**SD rat** **/F**	Hyperosmotic dextran solution or FGF-2 solution	Intraventricular injection	SA 12d	Communicating hydrocephalus model	Increased osmotic load on the ventricles could lead to hydrocephalus	[156]
	**Fischer rat/M**	200 μL autologous blood with either anti-CD47 blocking antibody (10 μg/mL) or IgG (10 μg/mL)	Intraventricular injection	A 1d-3d	Anti-CD47 blocking antibodies could relieve hydrocephalus	Endogenous phagocytosis might be a potential therapeutic strategy	[157]

Abbreviations: F, Female; M, Male; A, Acute hydrocephalus; SA, Subacute hydrocephalus; C, Chronic hydrocephalus; d, Day; w, Week; m, Month; E, Embryonic day; P, Postnatal day; VD, Ventricular dilatation; ICP, Intracranial pressure; CSF, Cerebrospinal fluid; VEGF, Vascular endothelial growth factor; CP, Choroid plexus; UPRmt, mitochondrial unfolded protein response; MC, Minocycline; sFRP-l, Secreted frizzled related protein-1; AN, Aminonicotinamide; FGF, Fibroblast growth factor; TRPV4, Transient receptor potential vanilloid 4; NKCC1, Na^+^/K^+^/2Cl^-^ ion co-transporter.

### 3.1.3 Other acquired hydrocephalus models

Other acquired hydrocephalus models are primarily based on CSF pathway obstruction, red blood cell lysis, and osmolarity alterations [86, 153, 156]. The details of these models are summarized in Table 2. Kaolin is the most widely used material for inducing acquired hydrocephalus in animals like neonatal or infant mice, rats, rabbits, cats, dogs, and sheep, owing to its simplicity and low cost. The injected kaolin diffuses into the subarachnoid space, causing inflammation and scar formation, leading to obstruction of the CSF pathways and eventually inducing ventricular enlargement. The main drawbacks of the kaolin model are that it may not accurately simulate the physiological and pathological changes of human PHH. Because the inflammation caused by kaolin can obstruct CSF production in the fourth ventricle, and it may be confused with the reaction of small glial cells in hydrocephalus, making it difficult to independently study the dynamic changes related to hydrocephalus [107]. The obstructive materials used to induce hydrocephalus should be chosen based on the criterion of inducing the mildest inflammation or histopathological changes possible. Second, this model has a high mortality rate and is prone to cause neural damage [152]. Intraventricular injection of exogenous peroxiredoxin-2 (Prx2) alone in rats, an important protein and pro-inflammatory factor in red blood cells, significantly expands the ventricles within 24 hours, while co-injection of Prx2 with clodronate sodium liposomes significantly reduces Prx2-induced ventricular enlargement [158, 159]. The mechanism may involve hydrocephalus induction through choroid plexus inflammation and ependymal membrane damage [160]. This also inspires the exploration of potential targets for hydrocephalus treatment, such as blocking the complement cascade associated with red blood cell lysis and improving hematoma clearance. There is also clinical evidence suggesting that osmotic pressure may play a role in the occurrence of hydrocephalus, but there is little research on this mechanism. In 2009, Krishnamurthy, S. et al. attempted to induce hydrocephalus by changing the osmotic load of CSF and believed that the osmotic load of the ventricles determines the water content of CSF [156]. They successfully induced communicating hydrocephalus by continuously injecting hyperosmolar solutions of dextrans and fibroblast growth factor-2 (FGF-2) into the lateral ventricle for 12 days [156]. However, the increased osmotic pressure would decrease over time, and further research is needed to determine whether it can maintain hydrocephalus [156]. Although not mainstream, these models are valuable for validating and reevaluating hydrocephalus pathogenesis. The therapeutic targets implicated in these models may also serve as promising avenues for further research.

### 3.2 Insights gained from genetic/congenital hydrocephalus models

Genetic hydrocephalus models and congenital hydrocephalus models refer to genetically engineered animal models and spontaneously mutated animal models with stable phenotypic inheritance [161] (Table 3). In rats, dominant genetic models of congenital hydrocephalus include the Hydrocephalus Texas (H-Tx) rat, the Wistar-Lewis (LEW/Jms) rat, and the 6-aminonicotinamide (6-AN) rat model of hydrocephalus. The H-Tx rat, developed in 1972, exhibits congenital obstructive hydrocephalus in the late fetal and perinatal stages, mirroring the late stages of human brain maturation during pregnancy [8, 162]. This strain has a predictable phenotype with aqueductal obstruction, increased intracranial pressure, ventricular enlargement and brain damage [163, 164]. The H-Tx strain, inherited in an autosomal recessive manner with environmental influences, is valuable for studying chronic hydrocephalus subtypes surviving months to years [8, 165]. The LEW/Jms rat strain, developed in 1983, is a model for studying neonatal hydrocephalus with prenatal aqueductal stenosis and hydrocephalus with a dominant disease incidence of 27.7% [163, 166]. However, the genetic causes of H-Tx and LEW/Jms rats remain unclear, raising concerns about their applicability. In addition, the 6-AN-related rat hydrocephalus model has morphological similarities to Dandy-Walker syndrome [167]. Spontaneously hypertensive rats (SHR), commonly used in hemorrhage studies, develop progressive ventricular enlargement at 4-8 weeks of age, although the mechanisms underlying hydrocephalus in SHR remain unclear [168]. In mice, widely used models include SUMS/NP, hydrocephalus-1 (hy-1), hydrocephalus-2 (hy-2), hydrocephalus-3 (hy-3) and L1CAM mutants [169, 170]. SUMS/NP mutant mice possess an autosomal recessive gene for congenital hydrocephalus, exhibiting early-onset aqueductal defects in hydrocephalus development. The hy-3 hydrocephalus mouse model presents aqueductal stenosis associated with a missense mutation in Hydin [171, 172]. However, the genetic causes for most strains remain unclear, raising concerns about their reliability.

**Table 3:** Genetic/congenital hydrocephalus models.

Gene/Strains	Species	Manipulation method	Features	Conclusion	Ref.
**Hyd rat**	Rat	-	Communicating hydrocephalus model	Inheritance of hydrocephalus in Hyd rats was dominant.	[173]
**H-Tx rat**	Rat	-	The H-Tx strain followed an autosomal recessive inheritance pattern with additional environmental influences	It was a valuable model for studying chronic subtypes of hydrocephalus that survive for months to year	[174, 175]
**LEW/Jms rat**	Rat	-	Hydrocephalus inheritance in LEW/Jms lines might be semidominant or involve multiple genes	Aqueduct obstruction might be the primary mechanism of hydrocephalus in LEW/Jms rats	[176, 177]
**Nme7**	SD rat	Knockout	All knockout rats developed hydrocephalus	Knockout of the NME7 gene mediated ependymal cell dysfunction	[178]
**Ccdc151**	C57BL/6N mouse	Knockout	A model of hydrocephalus derived from the primary ciliary dyskinesia model	CCDC151 gene was specifically expressed in ependymal cells of the ventricular system	[179]
**l1camb**	Zebrafsh larvae	Knockdown	It was similar to L1cam knockout mice with human L1 syndrome	L1CAM-mediated neurodevelopmental processes were regulated by protein domains	[180]
**Tmem67**	Wpk rat	Knockout	Communicating hydrocephalus model	TRPV4 antagonist inhibited progression of hydrocephalus	[181]
**Aqp4**	CD1 mouse	Knockout	Progressive, obstructive hydrocephalus model	AQP4 promoted transparenchymal-mediated CSF absorption	[182]
**Foxo3a**	Mouse	Knockout	Obstructive hydrocephalus model	The mechanism was the inactivation of the antioxidant defense pathway	[183]
**Piezo1**	Mouse	Knockout	Piezo1 may be a potential therapeutic target for hydrocephalus	This model caused abnormal accumulation of CSF by disrupting the development of meningeal lymphatic vessels	[184]

Abbreviations: TRPV4, Transient receptor potential vanilloid 4; AQP, Aquaporin; CSF, Cerebrospinal fluid.

Genetic hydrocephalus models are newly created strains developed through gene editing technologies such as CRISPR/Cas9 or gene knockout techniques involved in ventricular phylogeny and CSF circulation. The advantages of animal models for hereditary hydrocephalus lie in their natural occurrence of ventricular enlargement without the need for ventricular injections. These models exhibit distinct phenotypes, offer rich datasets, facilitate pathological comparisons, and allow for behavioral testing at a lower cost [8]. However, without shunting implements, they may die at a young age and are not suitable for long-term experiment. For instance, mutations in the neural cell adhesion molecule *L1cam* (L1) are a common pathogenic cause of X-linked hydrocephalus (XLH) in humans [10]. Interactions between *L1cam* and the ciliary morphology-related gene *Ccdc39* were explored, with *L1cam^y/-^* mutants in combination with *Ccdc39^prh/prh^* double mutants displaying more severe hydrocephalus, accelerating neonatal hydrocephalus development [185]. This suggests the importance of further investigating neocortical neural development [185]. McCarthy et al. developed a double-transgenic mouse model using the hGFAP promoter and the tet-off system, allowing control of *Ro1* expression levels the timing and rate of hydrocephalus development using doxycycline [186]. This model, the first to explore the relationship between astrocytic Gi signaling pathways and hydrocephalus, exhibited non-communicating hydrocephalus in all double-transgenic mice by postnatal day 15. The mechanism involves the restricted expression of *Ro1* in astrocytes altering the extracellular matrix, allowing more fluid to enter the ventricles, and defective adhesion molecules [8]. Such switchable and adjustable models of hydrocephalus mechanisms may provide more value for the study of its pathophysiology. Recently, numerous novel hydrocephalus models have emerged. For instance, a model successfully induced hydrocephalus in mice by knocking out *Piezo1* associated with the development and normal function of meningeal lymphatics, thereby impairing CSF drainage [184]. This innovation will inspire the development of more novel hydrocephalus models about lymphatic mechano-transduction, and we look forward to further breakthroughs in this area.

**Figure 2.**
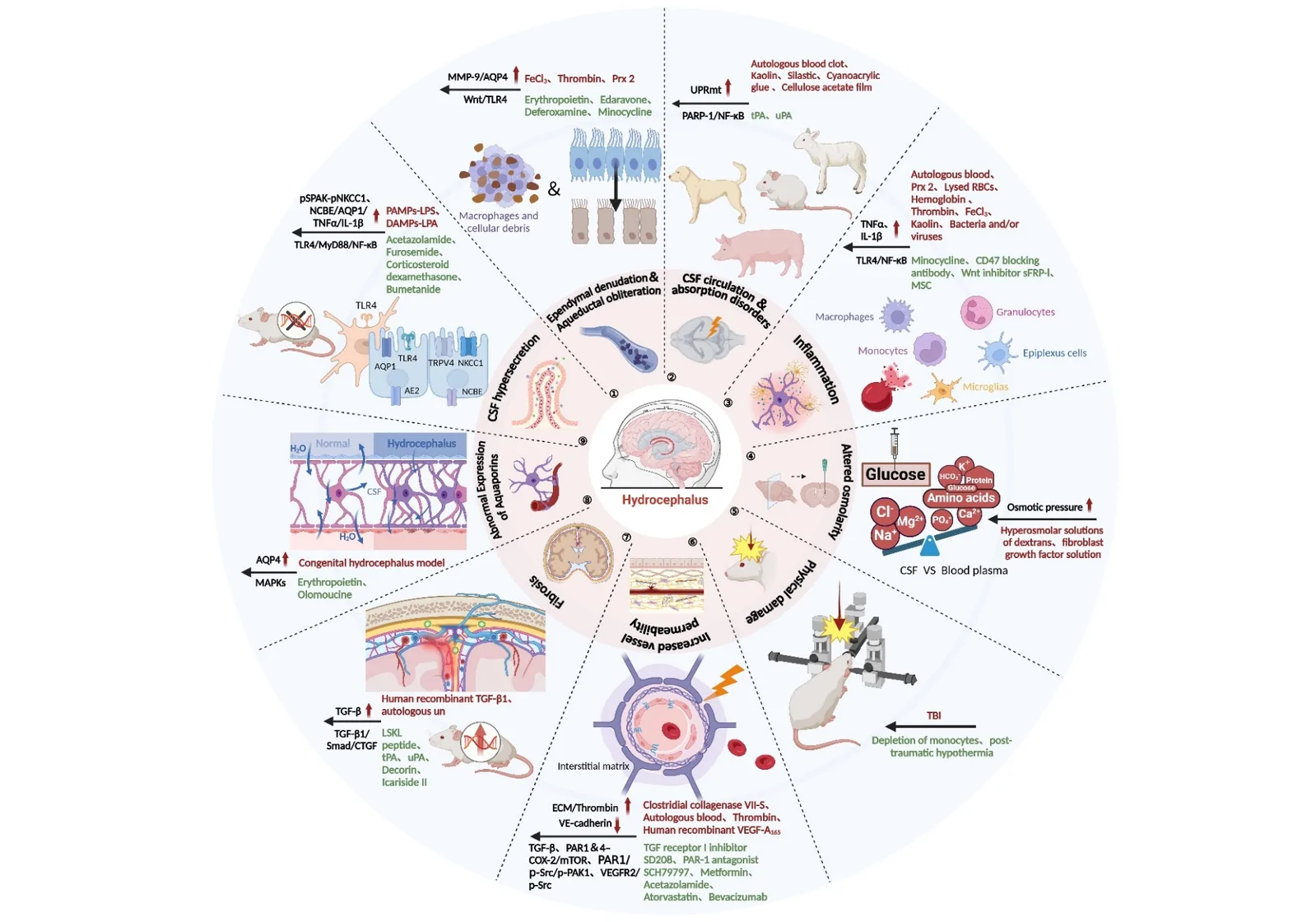
**Mechanisms in animal models of hydrocephalus Current models are mainly based on nine mechanisms: (1) Ependymal denudation and aqueduct obliteration: Blood components such as iron can be utilized to induce ependymal denudation, macrophage activation and neutrophil recruitment leading to hydrocephalus**. Iron overload is associated with MMP-9 hyperactivation and increased expression of AQP4. (2) CSF circulation and absorption disorders: Obstruction of the cerebral aqueduct or arachnoid villi caused by blood clots or microthrombi following hemorrhage can impair normal CSF circulation and reabsorption leading to hydrocephalus, which is associated with activation of microglia and UPRmt. (3) Inflammation: Following intraventricular hemorrhage, red blood cells release neurotoxic compounds triggering an inflammatory response. (4) Altered osmolarity: The production of CSF is closely related to the osmotic gradient between serum and CSF. Intraventricular injection of fibroblast growth factor solution or hyperosmolar dextran solution in rats can increase the osmotic pressure of CSF, leading to hydrocephalus. (5) Physical damage: Brain injuries can be induced in animal models through focal impact, diffuse impact, or a combination of both. Monocyte depletion and post-traumatic brain hypothermia has been shown to reduce tissue atrophy and retard the progression of PTH. (6) Increased vessel permeability: Both collagenase and thrombin can induce hydrocephalus by disrupting the integrity of blood vessels. (7) Fibrosis: Fibrosis may obstruct the circulatory pathways and reabsorption of CSF. Overexpression of TGF-β1 induced severe hydrocephalus in mice. (8) Abnormal expression of aquaporins: AQPs, water channel proteins related to the glymphatic system, have garnered attention in the field of hydrocephalus pathophysiology. (9) CSF hypersecretion: Experiments have shown that increased secretion of CSF promotes the occurrence of hydrocephalus. Red lettering indicates ingredients used to induce experimental hydrocephalus and green lettering indicates ingredients used to treat experimental hydrocephalus. Abbreviations: CSF, Cerebrospinal fluid; TBI, Traumatic brain injury; MMP-9, Matrix metalloprotein-9; AQP, Aquaporin; UPRmt, Mitochondrial unfolded protein response; PTH, Post traumatic hydrocephalus; NCBE, Sodium-driven chloride bicarbonate exchanger; AE2, Anion exchange protein 2; TRPV4, Transient receptor potential vanilloid 4; NKCC1, Na+/K+/2Cl- ion co-transporter; ECM, Extracellular matrix; VE-cadherin, Vascular endothelial-cadherin; MSCs, Mesenchymal stem cells; uPA, urokinase plasminogen activator; tPA, tissue plasminogen activator; PAR1, Protease activated receptor 1; PAR4, Protease activated receptor 4; p-Src, phosphorylated Src; p-PAK1, phosphorylated PAK1; COX-2, Cyclooxygenase-2; mTOR, mammalian target of rapamycin; TGF-β, Transforming growth factor-β; AQP, Aquaporin; SPAK, STE20/SPS1-related proline/alanine-rich kinase; TLR4, Toll-like receptor 4.

### 3.3 Contribution to understanding molecular and cellular mechanisms

The pathogenic mechanisms underlying various hydrocephalus models provide a foundation for developing personalized treatments tailored to distinct types of hydrocephalus. In this chapter, we summarize these mechanisms in detail. The primary mechanisms can be broadly categorized into nine types: (1) ependymal denudation and aqueduct obliteration, (2) CSF circulation and absorption disorders, (3) inflammation, (4) altered osmolarity, (5) physical damage, (6) increased vessel permeability, (7) fibrosis, (8) abnormal expression of aquaporins, and (9) CSF hypersecretion (Fig. 2).

### 3.3.1 Ependymal denudation and aqueduct obliteration

**Figure 3.**
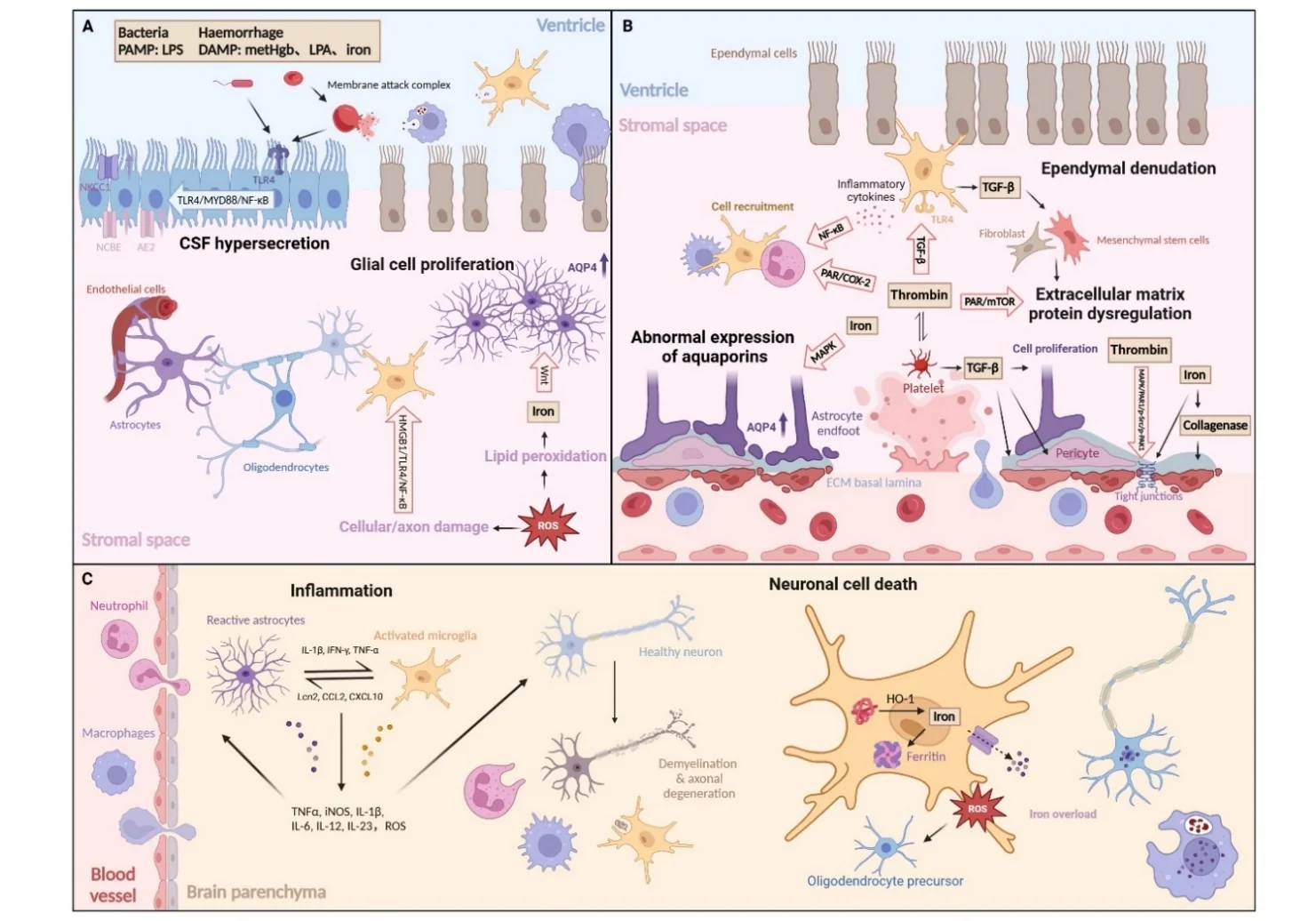
**The pathophysiology of common hydrocephalus models**. (**A**) DAMPs or PAMPs bind to TLR4 on CPe cells or microglia, producing pro-inflammatory cytokines and activating ion transporters such as NKCC1, NCBE and AE2. This leads to a net increase in CSF production. Free radical damage and the membrane attack complex mediate the spontaneous lysis of unphagocytosed red blood cells, releasing substances such as iron. Iron overload generates ROS, inducing cellular/axonal damage and lipid peroxidation, causing oxidative stress damage to ependymal cells. Lipid peroxidation products activate the WNT/β-catenin pathway, leading to the activation of reactive astrocytes and microglia during hydrocephalus, enhancing the fibrotic process. (**B**) Thrombin can elevate COX-2 and mTOR signaling pathways via PAR1 & 4 following IVH, inducing dysregulation of extracellular matrix proteins and hydrocephalus. It also induces significant expression of TGF-β1 and fibrosis. Furthermore, accumulation of iron around the ventricles leads to abnormal expression of aquaporins, further causing disturbances in water transport across the choroid plexus or disruption of choroid plexus integrity. Iron overload also results in the excessive activation of matrix metalloproteinases and degradation of tight junction proteins, inducing the breakdown of the blood-brain barrier, thereby promoting the occurrence of hydrocephalus. (**C**) Activated microglia and astrocytes collaborate to secrete pro-inflammatory cytokines, infiltrating the brain and causing neurotoxic damage, thereby amplifying inflammation and promoting the formation of glial scarring. Additionally, iron overload from hemolytic byproducts generates ROS, leading to periventricular white matter damage, which facilitates the progression of hydrocephalus. Phagocytes processing hemolytic debris exhibit increased phagocytic activity, which in turn heightens oxidative stress. OPCs are particularly susceptible to oxidative stress, leading to apoptosis. Chronic hydrocephalus is also characterized by cortical neuronal apoptosis, indicating widespread cellular damage and degeneration associated with sustained inflammation and oxidative stress. Abbreviations: CSF, Cerebrospinal fluid; SVZ: Subventricular zone; TLR4, Toll-like receptor 4; NCBE, Sodium-driven chloride bicarbonate exchanger; AE2, Anion exchange protein 2; ROS, Reactive oxygen species; IVH, Intraventricular hemorrhage; PAR1&4, Protease activated receptor 1&4; COX-2, Cyclooxygenase-2; mTOR, Mammalian target of rapamycin; TGF-β, Transforming growth factor-β; ROS, Reactive oxygen species; AQP, Aquaporin; DAMPs, Damage-associated molecular patterns; PAMPs, Pathogen-associated molecular patterns; NF-κB, Nuclear factor-κB; MAPK, Mitogen-activated protein kinases; HMGB1, High mobility group box-1 protein; SPAK, STE20/SPS1-related proline/alanine-rich kinase; HO-1, Heme Oxygenase-1; NKCC1, Na^+^/K^+^/2Cl^-^ ion co-transporter; OPCs, oligodendrocyte progenitor cells.

Ependymal denudation is a characteristic feature of congenital hydrocephalus, validated in both human fetal cases and animal models [187, 188]. Ependymal cells form a ciliated monolayer on the surface of the ventricles, facilitating the flow of CSF. The subventricular zone (SVZ) contains a complex network of microvessels, microglia, astrocytes, and oligodendrocytes, with astrocyte end-feet enveloping blood vessels, thereby regulating the exchange between CSF and blood. Microglia serve as the resident immune cells of the central nervous system (Fig. 3A). In conditions associated with PHH and PIH, red blood cells or lipopolysaccharides entering the CSF rapidly activate microglia residing in the choroid plexus to perform phagocytosis [3]. In rodent models of PIVH, oxidative stress, evident by increased malondialdehyde (MDA) and decreased superoxide dismutase (SOD) levels, is observed in the cortex and hippocampus [189]. Iron derived from hemorrhage triggers oxidative stress that damages ependymal cilia, disrupting normal CSF flow and contributing to ependymal denudation [189, 190]. In an inflammatory environment, the integrity of the ependymal lining can be compromised, allowing activated monocytes/macrophages to migrate into the ventricular system to clear blood cells and other debris [7, 24]. This phenomenon may facilitate the infiltration of blood components into the periventricular area, disrupting neurogenesis and the migration of new neurons, leading to secondary injury and accelerating the deterioration of hydrocephalus [7, 87, 191-193]. Over time, denuded areas are infiltrated by reactive astrocytes, known as gliosis [8]. Following ependymal denudation, the accumulation of cellular debris, recruited glial cells and macrophages, red blood cells, and other blood components can lead to aqueduct obliteration [24, 42, 76, 158, 194]. The denudation of ependymal cells also signifies the loss or dysfunction of cilia, which hampers CSF flow, thereby exacerbating the risk of aqueduct obliteration [8, 24].

Accordingly, several drugs with free radical scavengers, such as Edaravone, can alleviate hydrocephalus and nervous system damage by protecting the ependyma and neurons,which is in part mediated by inhibiting Keap1 expression and facilitating Nrf2 stabilization and accumulation [189]. Additionally, Minocycline emerges as a promising therapeutic candidate for hydrocephalus due to the drug’s direct anti-inflammatory, anti-oxidative, and neuroprotective properties [160].

### 3.3.2 CSF circulation and absorption disorders

Obstructive/non-communicating hydrocephalus occurs when the flow of CSF is blocked in the narrow apertures connecting ventricles. It happens quite often in patients post hemorrhage due to the formation of blood clots or microthrombi following bleeding, which may block the cerebral aqueduct, and the CSF outflow channels in the subarachnoid space. Based on this mechanism, many hydrocephalus models are created by injection of obstructive materials, such as kaolin, silastic or cyanoacrylic gel glue into cisterns or subarachnoid space (Table 2) [150, 195-197]. Jiebo Zhu and colleagues presented evidence that microglial activation and mitochondrial unfolded protein response (UPRmt) are key features in a kaolin-induced mouse model of hydrocephalus, linking these processes with the activation of PARP-1/NF-κB signaling and apoptotic cell death [151].

In this model, kaolin injection into the brain leads to ventricular enlargement and subsequent hydrocephalus, triggering an inflammatory cascade primarily mediated by microglial cells [151, 198]. Microglial activation is accompanied by an upregulation of UPRmt, a stress response that attempts to restore mitochondrial function in damaged cells. However, prolonged UPRmt activation can lead to cellular stress and damage when compensatory mechanisms are overwhelmed. The involvement of PARP-1/NF-κB signaling further amplifies the inflammatory response. PARP-1, an enzyme involved in DNA repair, is activated under conditions of oxidative stress and cellular damage, contributing to the transcriptional activation of NF-κB—a key regulator of pro-inflammatory cytokines [151, 198-200]. This signaling cascade promotes inflammation and triggers apoptotic pathways in susceptible cell populations, exacerbating neurodegeneration and glial scarring in hydrocephalus.

Meanwhile, drugs with thrombolytic therapeutic effect, e.g. urokinase plasminogen activator (uPA) and tissue plasminogen activator (tPA) may be potential treatment for obstructive hydrocephalus, partially validated by Gaberel et al. [201].

### 3.3.3 Inflammation

Inflammation, including acute reparative inflammation and chronic pathological inflammation, is a crucial component of pathogenesis of hydrocephalus [3]. Following intraventricular hemorrhage, red blood cells are lysed in the CSF, releasing neurotoxic compounds such as hemoglobin, heme, and iron, triggering an inflammatory response [87, 202]. Microglia and astrocytes are activated, recruiting phagocytes or monocytes and neutrophils by secreting pro-inflammatory cytokines [203]. Microglia, macrophages, oligo-dendrocytes, and ependymal cells act as iron scavengers, preventing the spread of iron to other parts of the brain [7]. When the iron released by hemorrhage exceeds the binding capacity of these cells, iron overload induces apoptosis and periventricular white matter damage through oxidative stress. Iron overload leads to the production of reactive oxygen species (ROS), which initiate lipid peroxidation and damage cellular structures, particularly in ependymal cells lining the ventricular system. This cellular damage triggers the release of damage-associated molecular patterns (DAMPs) that activate toll-like receptor 4 (TLR4) pathways on microglia and astrocytes. TLR4 activation stimulates a cascade of downstream inflammatory signals, including nuclear factor-kappa B (NF-κB) and mitogen-activated protein kinase (MAPK) pathways, which amplify the inflammatory response and promote the release of additional cytokines [3, 158, 204-206]. These molecular signals not only drive chronic inflammation but also foster a gliotic response characterized by the proliferation of reactive astrocytes and microglia, which further disrupts normal CSF flow and clearance, exacerbating hydrocephalus pathology [3]. This iron-induced oxidative stress also disrupts the BBB, a critical event that contributes to the chronic inflammatory environment associated with hydrocephalus. Over time, chronic inflammation leads to persistent immune cell infiltration, BBB breakdown, and fibrosis in the periventricular regions, forming gliotic scars that hinder normal brain architecture and CSF dynamics [3, 203, 207]. This sustained inflammatory state perpetuates a cycle of neurotoxicity, cellular damage, and CSF flow impairment, establishing a pathological foundation that promotes and exacerbates hydrocephalus [203] (Fig. 3C). Reducing the secretion of pro-inflammatory cytokines, depleting macrophages, microglia, and neutrophils, or inhibiting their activation, provides a therapeutic option for hydrocephalus, e.g., CD47-blocking antibodies, mesenchymal stem cell transplantation, and minocycline [98, 159, 160, 208, 209]. However, not all anti-inflammatory drugs are effective, and they may only be one pathway to alleviating hydrocephalus [209].

### 3.3.4 Altered osmolarity

The osmotic gradient between the apical and basolateral membranes of the choroid plexus epithelial cells is a crucial determinant of CSF circulation, directly influencing the water content of the CSF [24]. Studies have demonstrated that altering osmolarity, such as through intraventricular injection of hyperosmolar dextran or fibroblast growth factor can induce hydrocephalus by promoting fluid exchange between the ventricles and the choroid plexus or brain parenchyma [156].

### 3.3.5 Physical damage

Hydrocephalus happens quite often post-traumatic brain injury, known as PTH [122]. The focal impact or diffuse impact may lead to BBB disruption and brain tissue damage. Researchers have observed monocyte infiltration, cilia loss, and diminished CSF flow in animals with TBI-induced hydrocephalus [210, 211]. In mouse models, PARP-1 activation drives microglia from a resting to an activated state, leading to the release of various pro-inflammatory cytokines and cytotoxic substances, such as tumor necrosis factor-alpha (TNF-α) and interleukin-1 beta (IL-1β) [198]. These inflammatory mediators and cytotoxins exert direct and indirect neurotoxic effects, disrupting normal neuronal functions and ultimately inducing neuronal cell death. In the context of hydrocephalus, this microglial activation and cytokine release contribute to a neuroinflammatory environment that exacerbates ventricular enlargement and white matter damage [3, 198, 212]. The sustained release of these factors can disrupt cerebrospinal fluid flow by damaging the ependymal lining and impairing neural networks, thereby accelerating the progression of hydrocephalus. Monocyte depletion has been shown to reduce tissue atrophy and retard the progression of PTH [210, 213].

### 3.3.6 Increased vessel permeability

CSF is primarily derived from plasma filtrate entering the choroidal interstitium through the capillaries of the choroid plexus [24]. Therefore, disrupting endothelial tight junctions or degrading the extracellular matrix (ECM) to increase vessel permeability is a crucial approach in constructing hydrocephalus models. Vascular endothelial (VE)-cadherin, a component of adherens junctions in endothelial cells, plays a significant role in maintaining the integrity of the BBB. Following intraventricular hemorrhage, thrombin downregulates VE-cadherin levels in the choroid plexus, leading to increased vessel permeability and leakage of CSF via the PAR1/p-Src/p-PAK1 pathway, ultimately resulting in hydrocephalus [214] (Fig. 3B). Additionally, thrombin activation of the PAR1/COX-2 and PAR4/mTOR pathways further induces experimental hydrocephalus and disrupts the BBB by promoting extracellular matrix protein proliferation and exacerbating inflammation [192, 214, 215]. Matrix metalloproteinases (MMPs) comprise a family of intracellular peptide enzymes involved in tissue remodeling under various physiological conditions. Among them, collagenase is particularly noteworthy for its ability to catalyze the hydrolysis of collagen, a key component of blood vessel basement membranes. These intracellular proteases can be injected into the ventricles to degrade collagen-rich ECM basal lamina, thereby increasing BBB permeability and potentially inducing spontaneous hemorrhage, which contributes to the development of hydrocephalus [215]. Following SAH, pericytes secrete elevated levels of Cyclophilin A (CypA) [216]. This secretion activates the CD147 receptor and the downstream NF-κB pathway, which in turn induces the expression of matrix MMP-9 [216]. Consequently, this cascade leads to the degradation of endothelial tight junction proteins and the basement membrane, thereby compromising the integrity of the BBB [216].

Research has demonstrated that the severity of hydrocephalus can be mitigated by enhancing the expression of VE-cadherin, antagonizing VEGFR2, or inhibiting Src phosphorylation, as evidenced by the effects of Metformin [62]. Other research focuses on pharmacological agents that modulate genes and protein expression, such as inhibitors of matrix metallo-proteinases like MMP-9, which reduce periventricular tissue damage and improve hydrocephalus symptoms.

### 3.3.7 Fibrosis

Fibrosis occurs in several locations, including the periventricular region, brain parenchyma, subarachnoid space, and choroid plexus, which may obstruct both CSF flow and absorption. For instance, fibrosis of the periventricular region reduces its compliance, impairing the brain's ability to accommodate fluctuations in ICP. This reduced adaptability can exacerbate the compression of brain tissue, contributing to the progression and worsening of hydrocephalus. Similarly, fibrosis of the arachnoid granulations hinders CSF absorption, leading to its accumulation and further complicating the condition. Chronic inflammation-induced fibrosis involves increased local collagen synthesis, subependymal gliosis, and excessive deposition of ECM components [204, 217]. Following intraventricular hemorrhage, platelets and activated microglia release TGF-β molecules, which activate astrocytes, mesenchymal stem cells, and fibroblasts to synthesize and secrete large amounts of ECM proteins, such as collagen, hindering the drainage and exchange of interstitial fluid [218]. Elevated levels of TGF-β1 have been observed in both animal models and preterm infants with PHH [219]. By inserting a 1.35-kb porcine TGF-β1 cDNA into the glial fibrillary acidic protein (GFAP) gene via single-cell stage embryo injection, researchers have established a transgenic mouse model with high TGF-β1 expression in the brain. This model exhibits severe communicating hydrocephalus and upregulation of ECM proteins such as laminin and fibronectin [220]. MMP-9 and its specific inhibitor, TIMP-1, may contribute to the spontaneous development of hydrocephalus in this model by altering the ECM environment [221].

The TGF-β1/Smad/connective tissue growth factor pathway is recognized as a critical amplifier of profibrotic effects of TGF-β1 in various tissues. Consequently, competitive antagonists of TGF-β1, such as Decorin and the LSKL peptide, have demonstrated efficacy in preventing the progression of chronic hydrocephalus [219, 222]. Additionally, serine proteases that promote fibrinolysis, such as uPA and tPA, can mitigate fibrosis and thereby inhibit the development of hydrocephalus [223-225].

### 3.3.8 Abnormal expression of aquaporins

AQP4 and AQP1 are among the most extensively studied aquaporins, with AQP4 promoting CSF absorption during hydrocephalus events, and AQP1 serving as the basis for CSF secretion. The glymphatic CSF-ISF (interstitial fluid) exchange system represents one of the key pathways for CSF clearance. CSF from the subarachnoid space flows into the brain parenchyma along the Virchow-Robin spaces surrounding the arterial vessel, where it dynamically exchanges with ISF. It then exits the brain parenchyma through the Virchow-Robin spaces of venous vessel. This process is mediated by polarized AQP4 channels located on the endfeet of astrocytes surrounding both arteries and veins [24, 36, 226] (Fig. 3B). Studies have shown that mice with AQP4-deficient develop a more pronounced hydrocephalus phenotype more rapidly than wild-type mice, with reports suggesting that this deficiency results in approximately a ~70% reduction in interstitial solute clearance [182, 226]. AQP1 is expressed on the apical membrane of the choroid plexus and can leak into the CSF in cases of obstructive hydrocephalus in term-pregnancy infants, serving as a potential diagnostic biomarker [227]. In kaolin-induced hydrocephalus mouse models, AQP1-deficient mice exhibited a reduction in ventricular size compared to wild-type mice [228]. Oshio et al. created AQP1 knockout mice through targeted gene disruption, revealing that the absence of AQP1 resulted in a 25% reduction in CSF production [29].

Erythropoietin, a hematopoietic growth factor, can upregulate the expression of AQP4, reduce ventricular enlargement, and may serve as a potential therapeutic agent. Additional, the cyclin-dependent kinase inhibitor olomoucine improved the redistribution of AQP4 from paravascular regions to the stromal space following GMH, effectively alleviating hydrocephalus induced in the GMH model [226].

### 3.3.9 CSF hypersecretion

In certain cases, the body may attempt to compensate for excess CSF accumulation by increasing CSF absorption. Researchers have observed the growth of capacity for CSF clearance in hydrocephalic mice [202]. However, when this compensatory mechanism is insufficient to counteract the persistent overproduction of CSF, hydrocephalus will ultimately develop. Both experimental and clinical evidence support that CSF hypersecretion induced by inflammation of the choroid plexus promotes the occurrence of hydrocephalus [37]. Evidence from studies confirms that the CSF hypersecretion induced by intraventricular hemorrhage depends on the TLR4-NF-κB signaling pathway [37]. DAMPs and PAMPs bind to TLR4 receptors on the surface of choroid plexus epithelial cells or ependymal cells lining the choroid plexus, augmenting the activity of ion transporters such as NCBE, AE2 and NKCC1, thereby resulting in a net increase in CSF production [3, 37] (Fig. 3A).

Knockdown of the TLR4 or SPAK genes has been demonstrated to alleviate hydrocephalus [37]. NF-κB inhibitors, TLR4 inhibitors, NKCC1 blockers such as bumetanide, and anti-inflammatory drugs like dexamethasone have all been demonstrated to reduce CSF secretion [37, 229].

## 4. Current Treatment Strategies for Hydrocephalus

### 4.1 Surgical interventions in clinical practice

Currently, the treatment of hydrocephalus can be broadly categorized into surgical and pharmacological approaches, with surgery being the most common and effective method. The most prevalent surgical treatments include CSF shunt surgery and endoscopic third ventriculostomy (ETV).

### 4.1.1 Shunting treatment

Shunt surgery involves placing a proximal shunt catheter into the ventricular system or the cisterna magna to divert excess CSF to other areas of the body, such as the abdominal cavity, the pleura, or the atrium of the heart, thereby relieving ICP. Common shunt procedures include ventriculoperitoneal shunt (VP shunt), ventriculoatrial shunt (VA shunt) and lumboperitoneal Shunt (LP Shunt). Despite the maturity of these techniques and their efficacy in alleviating hydrocephalus symptoms, shunt implantation is still associated with failures, mechanical complications, and postoperative issues. For instance, the "overall shunt failure rate per year" for mixed hydrocephalus patients with ventricular frontal, occipital shunts, and cisterna magna shunts (CMS) was 9.0%, 12.6%, and 30.7%, respectively [230]. 40% of shunts need surgical revision within two years due to the risks of shunt blockage or rupture, regular monitoring and adjustments are required [231]. The infection rates for shunt procedures range from 0% to 35% depending on the study, and the reinfection rate following the first treatment of a shunt infection can be as high as 16%-26% [232-234]. Elderly patients face high postoperative complication risks, high readmission rates, and slow cognitive function recovery after surgery [235]. Although the LP shunt, which places a shunt in the lumbar region of the spinal cord, can reduce the risks and complication rates associated with brain surgery, it cannot be used to treat non-communicating hydrocephalus [41].

### 4.1.2 Endoscopic third ventriculostomy

ETV, a minimally invasive procedure, involves creating a small opening in the floor of the third ventricle using an endoscope to establish a direct CSF flow pathway from the ventricular system to the subarachnoid space, bypassing the obstructed area. It is mainly used for obstructive hydrocephalus and can avoid dependence on a shunt. ETV is often combined with choroid plexus cauterization (CPC) and is considered a better treatment option for elderly patients with normal pressure hydrocephalus (NPH) compared to VP shunting [236, 237]. However, it faces the risk of stoma closure due to gliosis and scar formation [238].

To address the risks and challenges associated with surgical treatment, researchers have proposed various strategies, including identifying biomarkers to predict shunt outcomes using machine learning algorithms, designing more efficient shunt devices such as programmable shunt valves and expandable catheters, and exploring alternative drainage locations. However, these strategies have not been particularly effective in mitigating post-shunt complications [239-243]. Studies have shown that many patients who undergo functional shunting continue to suffer from cognitive impairments and neurological deficits, underscoring the complexity of hydrocephalus pathophysiology. This suggests that in-depth exploration of the underlying mechanisms is key to developing preventive measures.

**Table 4:** Overview of current drug treatments for hydrocephalus.

Drug Category	Example Drugs	Clinical Indications	Benefits	Limitations	Ref.
**Thrombolytics / anti-fibrosis**	Urokinase, Streptokinase, tissue plasminogen activator	Post-hemorrhagic hydrocephalus, intraventricular hemorrhage	Facilitates clearing of intraventricular blood clots, potentially improving CSF flow	Risk of secondary bleeding, not suitable for non-hemorrhagic hydrocephalus	[244] [245, 246]
				Neurotoxic potential of tPA		
				May need to be combined with other treatments (e.g., shunting)		
**Anti-inflammatory Agents**	Dexamethasone, NSAIDs	Hydrocephalus due to inflammation, e.g., post-meningitis or traumatic brain injury	Reduces inflammation, decreases CSF production, alleviates edema	Does not address hydrocephalus' root cause, side effects such as immunosuppression, GI bleeding	[247-249]
			Short-term relief; temporary reduction of intracranial pressure	Chronic use risks may outweigh benefits, especially in elderly	
**Diuretics**	Mannitol, Furosemide, Acetazolamide	Acute ICP management, pre-surgery intervention for hydrocephalus cases	Rapid ICP reduction, temporary relief of symptoms	Ineffective for long-term treatment, risks of dehydration, electrolyte imbalances	[250-254]
			Useful when surgery is not immediately possible	Elderly patients may have more severe side effects (renal issues, heart failure)	
**Steroids**	Dexamethasone	Acute management of brain edema in hydrocephalus, inflammation control	Potent anti-inflammatory effects, reduces ICP and edema	Long-term use linked to immunosuppression, osteoporosis, no effect on chronic CSF dynamics	[229, 247, 255]

Abbreviations: ICP, Intracranial pressure; CSF, Cerebrospinal fluid.

### 4.2 Pharmacological approaches in clinical practice

In the pharmacological approaches of hydrocephalus, thrombolytics, diuretics and steroids are mainly used as adjuvant treatment methods to help relieve symptoms or reduce ICP before surgery. Elderly people are more prone to the side effects of drugs. Therefore, special attention needs to be paid to risk management, adjusting drug dosages, and closely monitoring the treatment process. Generally speaking, these drugs can only control the condition to a certain extent and are difficult to replace surgical operations, especially in the long-term treatment of hydrocephalus. We have summarized the pharmacological approaches of hydrocephalus in clinical practice (Table 4).

### 4.2.1 Thrombolytic agents

The enzymatic dissolution of intraventricular or subarachnoid blood accumulations and the inhibition of excessive extracellular matrix production represents potential therapeutic approaches for hydrocephalus. Clinically, thrombolytic agents such as urokinase-type plasminogen activator (uPA), tissue plasminogen activator (tPA), recombinant tissue plasminogen activator (rt-PA), and streptokinase are utilized to achieve enzymatic dissolution. These agents may reduce the likelihood of hydrocephalus development by interfering with inflammatory processes and extracellular matrix deposition. In a kaolin-induced communicating hydrocephalus rat model, uPA treatment significantly alleviated ventricular enlargement and inhibited the deposition of extracellular matrix molecules, such as laminin and fibronectin, in the subarachnoid space, while also attenuating gliosis [224]. In clinical settings, the intraventricular administration of tPA or rt-PA has shown potential in reducing shunt dependency. However, it may increase the risk of secondary intraventricular hemorrhage in infants and elevate the risk of ventriculitis in patients with ventricular drainage [256-258]. For elderly patients with fragile vasculature or altered coagulation function, careful risk assessment is essential before use.

### 4.2.2 Diuretics

Diuretics are commonly employed in the clinical management of hydrocephalus, functioning by drawing free water from tissues into the circulation, which is then excreted by the kidneys, or by reducing CSF production to alleviate hydrocephalus [259]. Isosorbide has shown promising effects in delaying or potentially avoiding shunt surgery for infants with hydrocephalus, serving as an adjunctive measure for temporary control of ICP prior to shunting. However, it is not recommended for treating hydrocephalus in children with spina bifida [260]. Other agents, such as glycerol, mannitol, and erythritol, have demonstrated efficacy in reducing CSF production and ICP in animal studies. Nonetheless, clinical research has yielded inconsistent results regarding their therapeutic efficacy.

Acetazolamide, a carbonic anhydrase inhibitor, has been shown to reduce CSF flow and ICP in animal models such as rabbits and cats, and improve clinical symptoms in dogs [261-263]. Clinical studies report that oral acetazolamide can lead to the complete resolution of hydrocephalus symptoms with good tolerance [264]. However, reports on pediatric cases vary, with some demonstrating gradual resolution of hydrocephalus, while others indicate no improvement or even increased neurological morbidity [253]. Furosemide has been observed to decrease CSF formation in rabbits and is often used in combination with acetazolamide. Still, its clinical efficacy in treating hydrocephalus remains controversial. Infants treated with high doses of acetazolamide and furosemide may develop nephrocalcinosis, prompting recommendations for close monitoring of urinary calcium excretion during treatment [251].

### 4.2.3 Steroids

Steroids, such as betamethasone and dexamethasone, are used primarily to mitigate the inflammatory response associated with hydrocephalus. Betamethasone has been shown to reduce CSF production by 43% in rabbits and can enhance intracranial compliance while inhibiting ICP elevation in hydrocephalus patients [265, 266]. Early studies on rabbits and dogs indicated that dexamethasone effectively reduces CSF secretion and suppresses ICP elevation [265, 266]. However, some research suggests that dexamethasone may not improve the progression of PHH in rat pups and could impair behavioral functions [267]. Despite these findings, most studies support its beneficial effects in human hydrocephalus patients [255, 268-270].

### 4.2.4 Others

Although some drugs for hydrocephalus treatment may only represent isolated cases, their relevance should not be underestimated. For instance, certain medications are utilized for infections related to CSF shunts or recurrent infections post-treatment. Studies have demonstrated that rifampin can reduce the risk of reinfection [271, 272]. Moreover, some drugs target functional impairments in hydrocephalus patients. In individuals with normal pressure hydrocephalus (NPH) who suffer from depression, methylphenidate, a drug that blocks dopamine and norepinephrine transporters, has been shown to improve both short-term and medium-term memory function [273, 274].Similarly, in young hydrocephalus patients with comorbid psychiatric disorders, the antidepressant trazodone has been reported to improve behavioral responses [275].

## 4.3 Innovations in treatment derived from animal research

### 4.3.1 Advances in surgical techniques

Animal research has been instrumental in advancing novel shunt systems and ETV. For example, to address the issue of proximal ventricular catheter (VC) obstruction, researchers tested a modified tethered liquid perfluorocarbon (TLP) VC in pig models. The study demonstrated that TLP VC could be a promising candidate for reducing proximal VC obstruction [276]. Other innovations include infection-resistant shunt coatings and shunt systems with auto-regulating valves, which have shown potential in reducing intracranial pressure fluctuations in animal studies. Prenatal ETV using a small rigid fetoscope has also been demonstrated as feasible in fetal lamb models with hydrocephalus [277]. Additionally, Jonghyun Oh et al designed a 10×10 microneedle array tested on pigs and it was found to effectively penetrate the dura mater, providing a CSF outlet without significant clogging [278].Despite recent innovations in surgical devices for hydrocephalus intervention, the inherent limitations, such as the complexity of long-term management and the persistent risks of infection and shunt obstruction, remain unresolved. Future research must focus on enhancing the performance of shunt systems, broadening the indications for surgical procedures, and leveraging emerging technologies, such as regenerative medicine, to address the fundamental challenges in surgical treatment.

### 4.3.2 Pharmacological innovations

Over the years, researchers have been exploring new pharmacological targets aimed at preventing and/or treating hydrocephalus (Table 5). Drugs tested in animal models for experimental hydrocephalus include aquaporin modulators, ion channel inhibitors, antioxidants, and agents targeting oxidative stress and iron overload. These therapies are evaluated both as monotherapies and in combination treatments [209]. For instance, erythropoietin (EPO), a neuroprotective agent, has been found to reduce ventricular enlargement in juvenile rats with obstructive hydrocephalus. Additionally, when used in combination with melatonin (MLT), EPO showed promise in preventing various pathological features of PHH in neonatal rats while reducing ependymal gliosis [279-281].

**Table 5:** Testing drugs in animal models prior to clinical trials.

Treatment strategies	Age	Animals	Models	Histological/biochemical change	Organic/functional change	Mechanism	Ref.
**uPA**	Adult	Rats	Other A-C	Inhibited the deposition of laminin and fibronectin, extracellular matrix molecules, attenuated gliosis, alleviate reactive astrocytosis, promoted the activation of HGF, down-regulated the TGF-β1 level	Improved learning and memory	Degraded fibrin and ECM components and promoted HGF release and activation	[224, 282]
**tPA**	Adult	Pigs	PIVH A-C	Preserved the integrity of the ependymal layer	Enhances the lysis of intraventricular blood clots	Removed intraventricular blood pharmacologically	[283]
**sFRP-l**	Adult	Rats	Other SA	Inhibited the expression of β-catenin and cyclin D-1 and alleviated reactive gliosis	-	Inhibited Wnt/β-catenin	[284]
**rhDecorin**	Adult	Rats	SAH C	Inhibited the expressions of TGF-β1/Smad/CTGF axis	Improved neurocognitive deficits	Suppresses extracellular matrix accumulation and following subarachnoid fibrosis via inhibiting TGF-β1/Smad/CTGF pathway	[285]
**Decorin**	Juvenile/3 week	Rats	Other SA	Increased in TGF-β1 and phosphorylated Smad2/3 levels, inhibited the deposition of the extracellular matrix molecules, laminin and fibronectin in the subarachnoid space; prevented astrogliosis	Protected against hydrocephalic brain damage, decreased myelin damage in the caudal internal capsule	Blocked TGF-β-induced subarachnoid fibrosis; prevented an increase in caudal periventricular white matter mean diffusivity as well as caudal corpus callosum axial and radial diffusivity	[286]
**Icariside II**	Adult	Rats	PIVH C	Reduced expression of members of the TGF-β1/Smad/CTGF signaling pathway	Improve long term neurocognitive deficits	Inhibition of the activating process of TGF-β1 and downstream Smad2/3 and CTGF signaling pathway	[204]
**SD208**	Neonatal/P7	Rats	GMH C	Decreased vitronectin and glial fibrillary acidic protein deposition	Improved cognitive and motor functions, and attenuated body weight loss	Inhibited TGF receptor I	[287]
**Ki16425**	Embryonic/E13.5	Mice	GMH A	Inhibited apical protrusions of ventricular cell clusters	-	Receptor antagonist against LPA1 and LPA3	[79]
**Memantine**	Neonatal/P7	Rats	Other C	Decreased astrocytic reaction in the corpus callosum, cortex and germinal matrix	Improved sensorimotor development, preserved spatial memory	N-methyl-D-aspartate receptor (NMDAR) antagonist	[288]
**LSKL peptide**	Adult	Rats	SAH C	Suppressed subarachnoid fibrosis	Improved long-term neurocognitive deficits	Inhibited TGF-β1 activity and subsequent Smad2/3 signaling, controlled the influx of calcium and promoting neuroprotection	[223]
**CD47-blocking antibodies**	Adult	Rats	PIVH A	Activated microglia/Monocyte-derived macrophage	-	Inhibited phagocytosis	[289]
**Minocycline**	Neonatal/P7	Rats	GMH A, Congenital C	Suppressed upregulation of ferritin, attenuated ventricular wall damage, as well as macrophage activation at the CP	Improved cognitive function and long-term motor function, white matter atrophy, and hippocampal neuronal cell loss	Iron-chelating activity,anti-inflammatory properties, a inhibitor of microglia and astrocytes activation	[98, 290]
**Edaravone**	Adult	Rats	PIVH A	Reduced ependymal cilia and neuron damage, inhibited oxidative stress, activated the Nrf2/HO-1 signaling pathway	Attenuated neurobehavioral disorder	Free-radical scavenger	[189]
**Monocyte depletion**	Adult	Mice	TBI SA	-	Preserved working memory, better motor coordination, and improved skill acquisition; enhanced preservation of functional white matter tracts	Abrogated circulating monocytes	[210]
**Erythropoietin + Melatonin**	Neonatal/P1	Rats	PIVH SA	Reduced excess ependymal GFAP expression; prevented Low YAP mRNA Levels; Ameliorated morphological damage to ependymal motile cilia	Prevented macrocephaly and neurodevelopmental delay	Prevent ependymal motile cilia damage	[291]
**Erythropoietin**	Infantile/2week	Rats	Other A	Increased expression of AQP4 in periventricular ependymal lining and cultured astrocytes and increased vascular formation, reduced denudation of ependymal line	Recovered body weight	Enhanced CSF resorption into the blood vessel by endothelial angiogenesis	[279]
**Melatonin**	Infantile/2week	Rats	Other C	Increased the tissue GSH level, abolished the increased levels of NO; ameliorated ratio of substantia grisea area/substantia alba area in the cerebellum	-	Antioxidant	[292, 293]
**recombinant human erythropoietin**	Adult	Rats	Other SA-C	Reduced astrocyte reactivity and microglial activation	Reduced progressive thinning of SVZ and proliferation rate	Inhibited lipid peroxidation, and reactive astroglIiosis	[281]
**Olomoucine**	Neonatal/P7	Rats	GMH C	Attenuated astrocytic proliferation	Improved short-term neurobehavior and long-term neurobehavioral function	Inhibition of the CDK	[226]
**Bumetanide**	Adult	Rats	PIVH A	-	Reduced CSF secretion	Inhibition of the Na^+^-K^+^-Cl^-^ co-transporter NKCC1	[37]
**STOCK1S-50699**	Adult	Rats	PIVH A	-	Reduced CSF secretion	Disrupt binding between SPAK and NKCC1	[37]
**Pyrrolidinedithiocarbamate**	Adult	Rats	PIVH A	Reduced p65 nuclear translocation; reduced abundance of CD68+ cells; Blocked upregulation of the pSPAK-pNKCC1 complex	Reduced CSF secretion	Inhibition of NF-kB signaling pathway	[37]
**Closantel**	Adult	Rats	PIVH A	-	Reduced CSF secretion	Inhibition of SPAK kinase activity	[37]
**TAK-242**	Adult	Rats	PIVH A	Abrogated activation of TLR4 signaling and activating phosphorylation of both SPAK and NKCC1	Reduced CSF secretion	Reduced CSF hypersecretion	[37]
**rh-IFN-α**	Neonatal/P7	Rats	GMH A	Increased phosphorylated JAK1, STAT1, and TRAF3; reduced phosphorylated NF-κB, IL-6, and TNF-α	Improved neurological functions, attenuated neuroinflammation, inhibited microglial activation	Activate the receptor-associated protein tyrosine kinases JAK1	[294]
**rh-relaxin-2**	Neonatal/P7	Rats	GMH A	Inhibited mast cell response	Improved neurological functions; restored cortical thickness and white matter area after GMH	Attenuated the degranulation of mast cells	[295]
**Dabigatran**	Neonatal/P7	Rats	GMH A	Reduced expression of short-term p-mTOR and long-term extracellular matrix proteins	Improved performances on the foot fault and rotarod tests	Anti-coagulant	[296]
**Acetazolamide**	Adult	Rats	PIVH A	-	Reduced CSF production	Carbonic anhydrase inhibitor	[63]
**Celecoxib**	Neonatal/P7	Rats	Other SA	Reduced neuroinflammation, and astrogliosis in different brain regions	Improves the neurobehavioral response	Selective COX-2 inhibitor	[64]
**Bevacizumab**	Adult	Rats	Other A	Inhibited ependymal cell denudation	-	VEGF inhibitor	[297]
**SCH79797**	Adult	Rats	PIVH A	Reversed the dampened VE- cadherin by inhibiting PAR1/p-Src/p-PAK1 pathway	-	Inhibited PAR1	[214]
**Atorvastatin**	Adult	Rats	ICH SA	Blocked neuron apoptosis, and decreased plasma MMP-9	Reduced the brain water content	Inhibited 3-hydroxy-3methylglutaryl-CoA reductases	[298]
**Deferoxamine**	Adult	Rats	TBI A, PIVH C	Attenuated heme oxygenase-1 upregulation; reduced iron levels and aquaporin-1 on the apical surface of the choroid plexus; suppressed both gene and protein expression of Wnt1 and Wnt3a in brain tissue	Improving performance on Water Morris navigation task	Iron chelators	[299, 300]
**Metformin**	Adult	Rats	PIVH SA	Increased VE-Cadherin Expression; diminished the Upregulation of VEGF Expression	-	Restrained VEGF signaling by antagonizing VEGFR2 or inhibiting Src phosphorylation	[62]
**Hyperbaric treatment**	Neonatal/P7	Rats	Other SA	Reduced glial fibrillary acidic protein	Improved agility and exploration of the environment, preservation of spatial memory, and greater learning capacity	Offered higher O_2_ rate to cerebral tissue	[137]
**Mesenchymal stem cell transplantation**	Neonatal/P4	Rats	PIVH A	Stimulate endogenous cell proliferation and differentiation	Improving functional sensorimotor outcome; Attenuated compression of the corpus callosum	Anti-inflammatory and anti-apoptotic	[208]
**Mesenchymal stem cell transplantation**	Neonatal/P4	Mice	PIVH SA	Improved the survival rates of EpPs and ependymal differentiation	-	Restored ependyma	[301]
**Unrestricted somatic stem cell injection**	Neonatal/3-4 hour	Rabbit	Other A-SA	Attenuated microglial infiltration; reduced apoptotic cell death, reactive astrocytes, and the levels of key inflammatory cytokines	Improved locomotor function	Anti-inflammatory	[100]
**Granulocyte-colony stimulating factor and lithium chloride**	Neonatal/P4	Rats	PIVH SA-C	Reduced neuronal apoptosis, improved expression of BrdU/GFAP, BrdU/NeuN and BrdU/PSA-NCAM	-	Inhibited neuronal apoptosis	[302]

Abbreviations: A, Acute hydrocephalus; SA, Subacute hydrocephalus; C, Chronic hydrocephalus; E, Embryonic day; P, Postnatal day; Other, Other acquired hydrocephalus models; ECM, Extracellular matrix; HGF, Hepatocyte growth factor; CSF, Cerebrospinal fluid; YAP, Yes-associated protein; GSH, Glutathione; LPA, Lysophosphatidic acid; GFAP, Glial fibrillary acidic protein; AQP, Aquaporin; NO, Nitric Oxide; sFRP-l, Secreted frizzled related protein-1; rhDecorin, recombinant human decorin; TGF-β1, Transforming growth factor-β1; JAK, Janus kinases 1; CDK, Cyclin-dependent kinase; COX, Cyclooxygenase; SPAK, STE20/SPS1-related, proline-alanine-rich kinase; JAK, Janus kinase; STAT1, Signal transducer and activator of transcription 1; TRAF3, Tumor necrosis factor receptor-associated factor 3; VEGF, Vascular endothelial growth factor; VE, vascular endothelial; SVZ, Subventricular zone; MMP, Matrix Metalloproteinase; PAR, Protease - Activated Receptor; NKCC1, Na^+^/K^+^/2Cl- ion co-transporter; EpPs, Ependymal progenitors.; BrdU, bromodeoxyuridine; NeuN, Neuronal nuclei; PSA-NCAM, polysialylated-neural cell adhesion molecule.

### 4.3.3 Stem cell therapies

Stem cell therapy is emerging as a promising avenue in hydrocephalus treatment, particularly in repairing neural damage. The view of pediatric hydrocephalus has shifted from a model of impaired CSF flow to a paradigm centered on dysregulated neural stem cell (NSC) fate [303]. Optimizing neural development has become a key strategy for treating hydrocephalus, especially in pediatric cases. In rodent and canine models, stem cell transplantation has been shown to repair damaged neural tissue, reduce inflammation, and promote CSF absorption. Studies have demonstrated that combining granulocyte-colony stimulating factor, a known stem-cell mobilizer, with lithium chloride significantly mitigated the development of post-hemorrhagic hydrocephalus in rats by inhibiting neuronal apoptosis [304]. Human umbilical cord-derived mesenchymal stromal cells (MSCs) have also shown neurogenic differentiation potential and migration capacity. Intracerebroventricular (IC) transplantation of MSCs significantly reduced PHH in neonatal rats, while intravenous (IV) transplantation may offer a less invasive option for clinically unstable, extremely preterm infants [208, 305]. Currently, IC transplantation of MSCs has shown promising results in phase I dose-escalation clinical trials in preterm infants [306].

### 4.3.4 Gene therapy

Gene therapy, as a highly precise therapeutic approach, offers transformative potential for the curative treatment of hydrocephalus by directly correcting pathogenic mutations or modulating the expression of disease-associated genes. This approach is particularly promising in cases of genetic or congenital hydrocephalus with well-defined molecular mechanisms. Over the years, studies utilizing genetic and congenital hydrocephalus models have provided a wealth of insights, positioning these models as a focal point in hydrocephalus research [307]. For instance, researchers have focused on *GemC1* and *McIdas*, two critical regulatory factors, to develop cell reprogramming-based therapeutic strategies aimed at converting specific cell types into ependymal cells. This approach seeks to repair damaged ependymal tissue, thereby restoring its functional integrity and improving the pathological conditions of hydrocephalus [308]. This approach aims to repair damaged ependymal tissue, restore CSF dynamics, and improve hydrocephalus outcomes. However, the current application of gene therapy remains largely limited to genetic forms of hydrocephalus or cases with clearly elucidated molecular mechanisms. Challenges persist in achieving efficient delivery to targeted sites within the central nervous system, ensuring long-term safety, and addressing the high costs associated with gene therapy development and implementation. Future advancements may lie in the integration of gene therapy with emerging neuromodulation technologies to develop multimodal therapeutic strategies. Such combined interventions could provide comprehensive solutions by addressing both the molecular and functional aspects of hydrocephalus, offering a promising direction for clinical and translational research.

## 5. Challenges to the Current Research Paradigms

Although animal models have provided valuable tools for investigating the pathophysiological mechanisms of hydrocephalus and developing therapeutic strategies, their limitations in anatomy, etiological complexity, and pharmacological effects must be fully acknowledged. Under the constraints of ethical and practical considerations, researchers should strive to optimize model design and incorporate multiple animal models to enhance the reliability and translatability of experimental findings.

### 5.1 Limitations of animal models in mimicking human hydrocephalus

Animal models have long been indispensable tools in biological and medical research. However, they frequently spark societal controversies and have notable limitations, including: (1) Species Differences and Translational Limitations: While there are notable similarities between humans and animals, fundamental anatomical and physiological differences between species can limit the applicability and predictive power of animal models in certain contexts. For example, the brain structures of mice and rats are relatively simple compared to humans, particularly in terms of ventricular anatomy and CSF circulation [161]. These differences hinder the accurate replication of the complex processes of CSF production, flow, and absorption in humans. Similarly, although zebrafish have proven advantageous in genetic research due to their transparency and rapid development, their CSF dynamics differ significantly from those of humans, limiting their relevance for studying certain aspects of hydrocephalus [309]. (2) Disease Complexity: Many human diseases involve intricate patho-physiological mechanisms that cannot be fully replicated in animal models. Hydrocephalus in humans is characterized by diverse etiologies such as hemorrhage, infection, tumors, or idiopathic causes. In contrast, most animal models rely on acute or artificially induced pathologies, such as genetic knockouts leading to hereditary hydrocephalus or chemical induction of acute hydrocephalus. These models often reproduce only isolated pathological features, failing to encompass the multifactorial and chronic nature observed in human hydrocephalus. Additionally, chronic hydrocephalus, which develops progressively over time and may involve long-term consequences, is particularly challenging to model in small animals due to their relatively short lifespans and the rapid induction methods typically used. Such methods may not fully reflect the temporal dynamics of chronic disease progression. For instance, slow ventricular enlargement, white matter degeneration, and long-term cognitive deficits, hallmarks of chronic hydrocephalus in humans, are difficult to assess in small animals like rodents and zebrafish. Furthermore, the simplistic physiological systems of small animals, while cost-effective and experimentally convenient, limit their clinical relevance for understanding the complexity of human hydrocephalus [310]. (3) Standardized approaches: A significant challenge in advancing animal models for hydrocephalus research lies in harmonizing diverse methodologies and effectively integrating the valuable preclinical insights they generate. For instance, TBI models, which are often used to study secondary hydrocephalus, face substantial variability in the methods used to simulate impact and the parameters applied. Variations in impact energy, duration, and site of injury can lead to inconsistent results, complicating the reproducibility and comparability of findings across studies. To address these issues, researchers have proposed incorporating interdisciplinary metrics to standardize experimental protocols. For example, quantifying tissue strain and strain rate offers a more precise and objective measure of biomechanical forces during injury [311]. These metrics could serve as a unifying framework to evaluate and compare the extent of brain injury and its subsequent effects on cerebrospinal fluid dynamics across different models and studies. By adopting such standardized approaches, the field could achieve greater consistency in preclinical findings, facilitating the translation of insights from animal models to clinical applications.

### 5.2 Ethical and practical considerations in animal research

The ethical and moral challenges surrounding the use of animals in research, particularly regarding animal welfare and rights, have long been a topic of significant concern. On one hand, animal experiments are subject to strict regulations and ethical review processes aimed at minimizing animal suffering. However, to alleviate pain and distress during experiments, animals are often administered anesthetics or analgesics. Research has shown that certain anesthetics, such as isoflurane and ketamine, possess neuroprotective or neurotoxic properties, which can introduce significant confounding effects, potentially biasing behavioral and histological outcomes [312]. On the other hand, large animals with greater anatomical and physiological similarity to humans, such as dogs, pigs, and non-human primates, offer higher translational relevance but face notable challenges. The costs associated with their housing, management, and experimentation are significantly higher, requiring specialized facilities and resources. Additionally, their use is subject to more stringent ethical scrutiny and societal opposition, particularly regarding non-human primates [313].

### 5.3 Translational challenges from animal models to human clinical practice

Translating findings from animal models of hydrocephalus to human clinical practice is a complex and challenging process, primarily due to the following factors. (1) Differences in clinical outcome assessments: Research using animal models typically focuses on structural and pathological changes associated with hydrocephalus, such as ventricular enlargement, expression of neuroinflammatory markers, neuronal apoptosis, and white matter demyelination [314-316]. These parameters provide crucial insights into the pathophysiological mechanisms of hydrocephalus. However, human clinical practice places greater emphasis on functional outcomes, including cognitive performance, motor abilities, quality of life, and the durability of symptom relief. For instance, patients with NPH often present with cognitive impairment, gait disturbances, or urinary incontinence—functional indicators that are challenging to replicate or directly assess in animal models. Furthermore, the evaluation methods commonly used in animal studies—such as measurements of ventricular size, histological staining, and molecular assays—are primarily focused on pathological changes and may not directly reflect improvements in clinically relevant outcomes. For example, observed structural improvements (e.g., ventricular reduction) may not necessarily translate into meaningful functional recovery for patients. In the evaluation of therapeutic strategies, animal studies often overestimate the potential effectiveness of treatments, as they lack rigorous validation of human-relevant functional outcomes. (2) Regulatory and translational gaps: The pathway from animal model research to clinical application often requires rigorous regulatory approval, a process that is inherently complex and challenging. One of the primary obstacles lies in the reproducibility and predictability of animal study results. Differences in research design, model selection, and evaluation methodologies across laboratories can lead to inconsistent conclusions. For example, there is a lack of standardized evaluation protocols for hydrocephalus therapies in animal models. Variations in the types of models used (e.g., genetic or acquired models), induction methods (e.g., chemical or physical), and assessment criteria complicate the establishment of a unified validation framework. These inconsistencies increase the difficulty of verifying therapeutic strategies before advancing them to clinical trials, potentially prolonging the development cycle for new treatments.

### 5.4 Differences in drug efficacy and safety between animal models and humans

The differences in drug efficacy and safety between animal models and humans present significant challenges in the clinical translation of hydrocephalus treatments. (1) Species differences and safety concerns: The practical challenges primarily stem from interspecies physiological differences and discrepancies in drug dosage and pharmacokinetics. Variations in the processes of drug absorption, distribution, metabolism, and excretion between animals and humans can lead to drastically different clinical outcomes in terms of drug efficacy and toxicity. For instance, a drug that shows potent therapeutic effects in animal models may exhibit reduced efficacy or increased adverse effects in humans due to distinct metabolic pathways [317]. This inconsistency underscores the critical importance of dosage adjustments and bioavailability studies during the translational process. (2) Lack of directly acting drugs: Currently, the development of clinical drugs with more direct mechanisms of action remains an unresolved issue. Although diuretics and corticosteroids, such as acetazolamide, furosemide, and dexamethasone, are sometimes effective, they are typically used as adjunct therapies and cannot control the long-term progression of hydrocephalus. Small molecule interventions, anti-inflammatory, and antifibrotic approaches are among the most common experimental treatments for hydrocephalus, but most of them have not become part of standard medical management due to safety concerns for clinical use [252, 318]. In conclusion, despite extensive research highlighting the critical roles of CSF production—such as the secretory activity of choroid plexus epithelial cells—and absorption, particularly via arachnoid granulations, in the pathogenesis of hydrocephalus, no clinically available drugs have yet been developed to significantly reduce CSF production or effectively enhance its absorption. This limitation primarily stems from an insufficient understanding of the regulatory mechanisms underlying these processes.

## 6. Future Directions and Research Priorities

### 6.1 Emerging technologies and their evaluation in animal models: Artificial intelligence, Neuroimaging

Emerging technologies such as artificial intelligence (AI) and advanced neuroimaging methods are reshaping the field of hydrocephalus research. AI can process vast biomedical datasets, enabling the quantitative assessment of ventricular volume, identification of hydrocephalus-related features, and elucidation of potential mechanisms and therapeutic targets through the integration of imaging data, clinical information, and patient biomarkers. Furthermore, AI-based predictive models can assess patient responses to shunt surgery, assisting clinicians in developing personalized treatment strategies. For example, AI can be combined with superb microvascular ultrasound to identify shunt malfunctions or assess radiological parameters, thus eliminating the chance of human error [319, 320]. And AI’s capability to analyze large, multi-dimensional datasets makes it invaluable for the discovery of biomarkers in hydrocephalus research. In animal models, AI-driven platforms can integrate data from multiple sources—such as genomics, transcriptomics, proteomics, and metabolomics—to identify molecular patterns that correlate with disease stages or therapeutic responses. This multi-omics approach can lead to the discovery of new biomarkers for early diagnosis, disease monitoring, or predicting therapeutic outcomes, which can then be validated in clinical settings. For example, machine learning algorithms can analyze gene expression profiles and identify genetic pathways that are significantly altered in hydrocephalus. These pathways can be explored in greater depth using animal models, where genetic manipulation or targeted interventions can validate the role of these biomarkers in disease progression or response to treatment. Neuroimaging techniques provide detailed observations of brain structure and CSF dynamics. High-resolution neuroimaging techniques, such as diffusion tensor imaging (DTI) and functional magnetic resonance imaging (fMRI), have been widely used in animal research. These techniques provide detailed information about the microstructural and functional changes in brain tissue. For instance, AQP4 has long been acknowledged for its significant role in maintaining brain water homeostasis. Its advancements in medical imaging and computational modeling related to CSF dynamics could provide a valuable framework for early diagnosis and prognosis in patients [321]. While AI and neuroimaging hold the potential to revolutionize hydrocephalus management and enhance patient care quality, challenges such as the "black box" nature of AI algorithms and the complexities surrounding the collection and sharing of neuroimaging data must be addressed.

### 6.2 Regenerative medicine and tissue engineering

Regenerative medicine and tissue engineering are becoming promising options for hydrocephalus treatment. The miniature biomimetic transdural shunt developed based on the bionic strategy has been reported to improve the ICP in elderly patients with hydrocephalus [322]. Cellular reprogramming and stem cell therapies have shown promising potential in hydrocephalus treatment. Stem cell approaches may promote neurogenesis in regions affected by hydrocephalus, with the possibility of repairing damaged ependymal tissues or ventricular structures through the integration of transplanted cells into existing neural circuits. Such integration holds the promise of significantly improving hydrocephalus management by restoring normal CSF dynamics and mitigating associated neurological damage. In addition, tissue-engineered constructs offer the potential to reconstruct or bypass obstructed CSF pathways, serving as alternatives to traditional shunting systems. For example, the development of biocompatible and durable materials capable of seamless integration with neural tissues, without triggering inflammation or fibrosis, could address the inherent limitations of shunting. Furthermore, engineered functional substitutes to enhance CSF absorption may address the absorption deficits often observed in hydrocephalus, thereby restoring the natural balance of CSF dynamics. Advanced drug delivery systems or engineered carriers targeting the choroid plexus could also offer personalized solutions for managing complex pathologies by modulating CSF production at its source. Integrating tissue engineering with CRISPR-based gene editing holds promise for achieving targeted interventions in hydrocephalus models. Smart biomaterials could be designed for the precise delivery of therapeutic molecules or genes to specific regions within the brain, enabling localized and controlled treatment.

### 6.3 Clinical management strategies for hydrocephalus

Traditional treatment methods for hydrocephalus often struggle to address the diverse clinical presentations and individual variability of patients, underscoring the limitations of a one-size-fits-all approach. As a result, there is increasing emphasis on precision medicine in hydrocephalus management to improve outcomes and enhance patient quality of life.

First, developing more efficient and reliable animal models is essential for understanding the mechanisms of hydrocephalus, identifying specific molecular targets, screening biomarkers, and initiating early-stage drug development. These models provide a controlled environment to investigate disease mechanisms and test potential interventions. The insights gained from animal studies must then be validated clinically, while clinical findings should, in turn, inform the refinement of animal models. This bidirectional integration is crucial. For instance, potential biomarkers and therapeutic targets identified in animal models need verification in patient populations to assess their specificity, stability, and predictive value across different subtypes of hydrocephalus. Clinical data, particularly genetic profiles, imaging results, and biomarker trends, can be fed back into animal research, guiding the modification of models to better mimic human conditions. For example, prevalent genetic mutations or clinical phenotypes observed in patients can drive the selection of animal models or influence genetic manipulations. Clinical failures can also help refine experimental design, avoiding redundant or ineffective approaches.

Second, to achieve precise diagnosis in hydrocephalus, a robust classification system integrating genomics, advanced imaging, and biomarker data is required [57, 323]. Genomic studies aim to identify high-risk genes and mutations associated with hydrocephalus. These findings can be translated into clinical practice through genomic sequencing, distinguishing patients based on genetic risks and developing risk prediction tools. Insights from these studies can be verified using gene-edited animal models, investigating the role of specific mutations in pathophysiological processes. High-resolution neuroimaging techniques such as MRI and CT are invaluable for assessing structural changes, CSF dynamics, and ventricular enlargement in hydrocephalus patients. These imaging findings can be compared with data from animal models, allowing the establishment of standardized diagnostic criteria. Functional imaging modalities like fMRI provide additional insights into the impact of hydrocephalus on neural function, which can be cross-validated with corresponding changes in animal models to ensure clinical relevance. Biomarker integration, including liquid biopsies from CSF and blood, allows for real-time patient monitoring. Tracking these markers dynamically could guide the timing of interventions and evaluate treatment efficacy.

Third, effective implementation of personalized therapy in hydrocephalus necessitates the involvement of a multidisciplinary team, including neurosurgeons, geneticists, neuroradiologists, and pediatricians. Utilizing genomic and phenotypic data can help predict individual responses to various therapeutic options. The establishment of electronic health records and comprehensive patient databases allows real-time monitoring and feedback, supporting data-driven adjustments in treatment strategies [324]. These platforms can integrate outcomes, biomarker shifts, and imaging data across patients, refining personalized approaches. Precision medicine emphasizes not only the initial diagnosis and treatment but also the continuous adjustment of therapeutic regimens. Ongoing follow-up can track disease progression, monitor treatment response, and enable timely modifications to the care plan. Additionally, the construction of data-sharing networks across healthcare and research institutions will facilitate the global exchange of genomic, imaging, and biomarker information. This broad-scale collaboration is vital for recognizing genetic variability across populations and enhancing the generalizability of precision therapies. The development of an effective precision medicine framework for hydrocephalus hinges on the synergy between basic and clinical research. As interdisciplinary cooperation and technological advances continue to evolve, this system will become increasingly refined, leading to improved individualized diagnostic and therapeutic strategies for hydrocephalus. Ultimately, such advancements will enhance patient outcomes and quality of life.

## 7. Conclusion

In conclusion, comprehensive advancements in hydrocephalus management rely on a synergistic understanding of its pathophysiology, the continuous refinement of animal models, and the translation of preclinical findings into effective clinical therapies. A clearer grasp of CSF dynamics and distinctions among hydrocephalus types has underscored the necessity of diverse animal models that range from rodents to larger animals. These models play a pivotal role in elucidating the cellular and molecular mechanisms behind hydrocephalus, thereby driving the development and validation of new therapeutic targets. Animal research remains central to the discovery and preclinical evaluation of surgical, pharmacological, and emerging molecular therapies. Innovations stemming from genetic and acquired models, including those exploring post-hemorrhagic and post-infectious hydrocephalus, are yielding insights that could transform treatment paradigms. Novel surgical approaches, gene therapy, regenerative medicine, and precision medicine are promising yet complex areas where animal studies are contributing critical data. However, the translational journey from animal models to human application is fraught with challenges, such as limitations in the models’ ability to replicate human pathology, ethical concerns, and discrepancies in drug responses. Addressing these translational hurdles and improving the fidelity of animal models are essential steps forward. Emerging technologies, including artificial intelligence and advanced neuroimaging, offer powerful tools to bridge knowledge gaps between animal research and clinical practice, enhancing model accuracy and treatment specificity. Looking ahead, a collaborative approach that integrates findings from both preclinical and clinical studies will be key to optimizing outcomes in hydrocephalus care, guiding the field toward more personalized, effective therapies.

## Supplementary Materials

The Supplementary data can be found online at: www.aginganddisease.org/EN/10.14336/AD.2024.1434.
