# Supplementary material for: Advancing Hydrocephalus Management: Pathogenesis Insights, Therapeutic Innovations, and Emerging Challenges
Source: Aging Dis. 2024 Dec 29;17(1):185–225. doi: 10.14336/AD.2024.1434 (PMC12727080; doi:10.14336/AD.2024.1434)
Supplement: Supplementary file 1 — The Supplementary data can be found online at: www.aginganddisease.org/EN/10.14336/AD.2024.1434. [file AD-17-1-185-s.pdf]

SUPPLEMENTARY DATA

Advancing Hydrocephalus Management: Pathogenesis Insights, Therapeutic Innovations, and Emerging Challenges

Xiuyun Liu, Hui Zhi, Marek Czosnyka, Chiara Robba, Zofia Czosnyka, Jennifer Lee Summers, Huijie Yu, Xiaoguang Tong, Guoyi Gao, Gelei Xiao, Kai Yu, Yan Xing, Renling Mao, Shaoya Yin, Yangong Chao, Hongliang Li, Ke Pu, Keke Feng, Meijun Pang, Dong Ming

© 2024. Liu X et al. Published online at http://www.aginganddisease.org/EN/10.14336/AD.2024.1434

SUPPLEMENTARY DATA

Methods

A comprehensive literature review was conducted by searching the Web of Science, PubMed, and Scopus databases for peer-reviewed English-language articles. The search terms included “hydrocephalus,” “cellular mechanism,” “treatment,” “animal models,” and “experimental hydrocephalus.” The review primarily focused on articles published within the past decades, that is, from 2014 to 2024, to ensure a thorough understanding of recent advancements. However, earlier seminal studies were also included to provide historical context and highlight the development of foundational concepts. The inclusion criteria encompassed experimental studies, clinical trials, and review articles directly relevant to the pathogenesis, treatment, and modeling of hydrocephalus. Particular attention was given to high-quality studies that provided significant contributions to understanding the disease mechanisms and therapeutic strategies. Early foundational studies were incorporated to highlight the development of key concepts, while recent publications were prioritized to present the latest advancements.

Supplementary Table 1. Animal models of Post - hemorrhagic Hydrocephalus

Type	Age	Species/Gender (F/M)	Injection of agent	Methods	Type of hydrocephalus	Features	Conclusion	Ref.
PIVH	Neonatal/P4	SD rat	200 µL of fresh maternal whole blood	Intraventricular injection	C 4w	Human umbilical cord blood-derived MSCs intervention	Intravenous administration of MSCs might be a promising treatment	[1]
	Neonatal/P8	C57BL/6J and Balb/c background mouse/-	LPA	Intraventricular injection	SA 1w	LPA1 and LPA3 were key mediators of PHH	The mechanism might be ventricular surface denudation and ciliary dysfunction, not physical obstruction	[2]
	Adult	SD rat/M	200 µL non-heparinized autologous blood	Intraventricular injection	A 1d-3d	The elevation of AQP4 level expression in the rat hydrocephalus model was verified	Downregulation of AQP4 expression could worsen hydrocephalus	[3]
		SD rat/M	130 µL of autologous vena caudalis blood/50 µL $FeCl_3$ (2 mmol/L)	Intraventricular injection	A-C 2d-4w	Iron activated the Wnt signaling pathway after IVH and regulated subarachnoid fibrosis	Edaravone alleviated hydrocephalus and neurological disorders by activating the Nrf2/HO-1 pathway	[4] [5]
		Wistar rat/M	200 µL homologous blood	Intraventricular injection	A 2d	The regulatory mechanism of CSF secretion was revealed	CSF hypersecretion was mediated by activation of the TLR4-dependent STE20-type stress kinase SPAK	[6]

© 2024. Liu X et al. Published online at http://www.aginganddisease.org/EN/10.14336/AD.2024.1434

		SD rat/M	200 μL homologous blood	Intraventricular injection	SA 1w	Metformin intervention	The occurrence of hydrocephalus was related to the activation of the VEGF/VEGFR2/p-Src pathway	[7]
		SD rat/M	50 μL $FeCl_3$ (2 mmol/L)/30μL lysed RBCs	Intraventricular injection	A 1d	Injections of packed RBCs could not cause hydrocephalus	Injection of iron resulted in VD and ependymal cell injury	[8]
		SD rat/M	200 μL of autologous blood/3 U thrombin	Intraventricular injection	A 1d	PAR-1 antagonist SCH79797 intervention	Thrombin-induced hydrocephalus was associated with ependymal damage and BBB disruption, which is mediated by PAR-1	[9]
		SD rat/M	3 U of rat thrombin	Intraventricular injection	A 1d	Carbonic anhydrase inhibitor acetazolamide or PAR1/p-Src/p-PAK1 inhibitors intervention	Thrombin down-regulated the expression of VE-cadherin in the CP	[10] [11]
		SD rat /F, M	5U/ 3U rat thrombin	Intraventricular injection	A 1d	Focusing on estrogen	Thrombin caused more severe VD and white matter damage in female rats	[12]
		SD rat /F, M	Recombinant rat Prx2 protein solution	Intraventricular injection	A 1d	MC intervention	Prx2 might cause hydrocephalus by inducing inflammation and damage to the ventricular wall	[13, 14]
		SD rat/M	LPA	Intraventricular injection	A 1d	TRPV4 was an ICP regulator that regulated the rate of CSF secretion	LPA directly acted on TRPV4 in adult rats to regulate NKCC1 and promote CSF hypersecretion	[15]
	Elderly	Fischer rat/M	50 μL $FeCl_3$ (2 mmol/L)	Intraventricular injection	A 1d	Minocycline or clodronate liposomes intervention	The stromal macrophages of elderly rats account for more than 10% of CP cells	[16]
GMH	Neonatal/E29	New Zealand white rabbit	50% glycerol: water (6.5 g/kg)	Intraperitoneal injection	A 1d	Unrestricted somatic stem cells intervention	Unrestricted somatic stem cells could exert anti-inflammatory effects to reduce	[17]

© 2024. Liu X et al. Published online at http://www.aginganddisease.org/EN/10.14336/AD.2024.1434

							pathological damage	
	Neonatal/2 h postnatal age	Rabbit pup/-	Glycerol	Intraperitoneal injection	SA 2w	It was difficult to replicate	Human pathological phenomena such as VD, glial cell hyperplasia, decreased myelination had been reproduced	[18]
	Neonatal/P7	SD rat/-	0.3 U of clostridial collagenase VII-S	Ganglionic eminence injection	C 4w	PPARγ stimulation and PPARγ antagonist intervention	PPARγ-induced upregulation of CD36 to promote hematoma lysis might be a key therapeutic target	[19]
		Rat/-	Bacterial collagenase	Ganglionic eminence injection	C 4w	COX-2 or mTOR inhibitors improved the hydrocephalus after GMH	Inflammatory and proliferative responses that might be upstream of dysregulation of extracellular matrix proteins	[20]
		SD rat/-	0.3 U of clostridial collagenase VII-S	Striatum injection	SA-C 1w-4w	Microglia and astrocyte activation inhibitor MC intervention	Treatment with MC reduced GMH-induced hydrocephalus and brain injury	[21]
		SD rat/-	0.3 U of clostridial collagenase VII-S	Ganglionic eminence injection	C 1m	Neurological consequences after GMH were described	This model corresponded to grades III-IV GMH in human preterm infants	[22]
ICH	Adult	Wistar rat/M	0.5 μL of collagenase (Type VII, 1μ/μL)	Caudate putamen injection	SA 7d	Atorvastatin intervention	Atorvastatin relieved hydrocephalus and inhibited neuronal apoptosis	[23]
		SD rat/M	200 μL autologous blood	Caudate nucleus injection/ Intraventricular injection	A-C 1d-4w	The ICH/IVH rat model resulted in the same bilateral VD, perhaps excluding interference with brain atrophy and tissue loss in assessing ventricular size	The ICH/IVH rat model induced more severe chronic hydrocephalus and cerebral iron deposition than the PIVH model	[24]
		SD rat/M	200 μL autologous blood	Caudate nucleus injection	A-C 1d-4w	Chronic hydrocephalus model	It was inconsistent with the high mortality rate in patients with ICH in clinical practice	[25]

© 2024. Liu X et al. Published online at http://www.aginganddisease.org/EN/10.14336/AD.2024.1434

SUPPLEMENTARY DATA

SAH-Injection	Neonatal/one-year-old	Cynomolgus monkey/F	2-3 mL of autologous blood	Cisterna magna injection	C 17d	Primate model animals	Extensive arachnoid granule fibrosis was present	[26]
	Neonatal/P10- P11	C57BL/6 mouse/-	300 ng of human recombinant TGF-β1	Parietal lobe injection	C 3w-6w	Slowly progressive communicating hydrocephalus model	Human recombinant TGF-β1 injection resulted in a reduction in cilia on the ependyma and deposition of collagen fibers in the leptomeninx intercellular space	[27, 28]
		CD-1 nude mouse/-	300 ng of human recombinant TGF-β1	Intraperitoneal injection	C 6w	Near-infrared fluorescence imaging methods	Near-infrared optical imaging could be used to monitor CSF movement	[29]
	Adult	SD rat/F	0.4 mL of non-heparinized autologous blood	Cisterna magna injection	C 2w	The expression of HGF and VEGF in hydrocephalus was analyzed for the first time	The pathological damage of chronic hydrocephalus after SAH was related to the high expression of HGF and VEGF	[30]
		SD rat/M	0.5 mL autologous un-heparinized blood	Cisterna magna injection	C 3w	Decreased expression of TGF-β1/Smad/CTGF signaling pathway was associated with chronic hydrocephalus and fibrosis	ICA II inhibited chronic hydrocephalus and subarachnoid fibrosis	[31]
SAH-Endovascular perforation	Adult	SD rat/M	-	Endovascular perforation technique	A-C 1d-23d	Elevated ICP was associated with VD and behavioral alterations	Rats with hydrocephalus suffered more severe hemorrhage and ventricular wall damage than rats without hydrocephalus	[32]
		SD rat/M	-	Endovascular perforation technique	A 1d	This model focused on changes in the cells of the plexus epiplexus cell	There was an increase in the number and size of epiplexus cells in rats with hydrocephalus, which may be related to thrombin	[33]
		SD rat /F, M	-	Endovascular perforation technique	A 1d	Gender influenced the development of acute	The incidence and severity of acute	[34]

© 2024. Liu X et al. Published online at http://www.aginganddisease.org/EN/10.14336/AD.2024.1434

						hydrocephalus after SAH	hydrocephalus in female rats were significantly higher than males	
TBI-Modified weight drop	Neonatal /P20	SD rat/M	-	The impact weight was allowed to fall freely	SA 2w	It was the first detailed study of intracellular electrophysiologic al changes after rmTBI	Compared to a single mTBI event, rmTBI could induce significant long-term VD	[35]
TBI-LFPI	Adult	SD rat/M	Lateral fluid percussion brain injury (2.5–3.5 atm)	-	A 1d	This study reported the acute hydrocephalus induced by the LFPI model	DFX treatment could reduce TBI-induced ipsilateral and contralateral VD	[36]
TBI-CCI	Adult	C57BL/6 mouse/M	CCI device with velocity of 2.5 m/s	-	SA 1w-2w	New therapeutic strategies for monocytes were presented	Depletion of monocytes attenuated hydrocephalus and preserved functional white matter	[37]

Abbreviations: F, Female; M, Male; P, Postnatal day; PIVH, Primary intraventricular hemorrhage; IVH, Intraventricular hemorrhage; GMH, Germinal matrix hemorrhage; ICH, Intracerebral hemorrhage; SAH, Subarachnoid hemorrhage; TBI, Traumatic brain injury; mTBI, Mild traumatic brain injury; rmTBI, Repetitive mTBI; LFPI, Lateral (parasagittal) fluid-percussion injury; CCI, Controlled cortical impact; A, Acute hydrocephalus; SA, Subacute hydrocephalus; C, Chronic hydrocephalus; h, Hour; d, Day; w, Week; m, Month; y, Year; VD, Ventricular dilatation; CSF, Cerebrospinal fluid; MSCs, Mesenchymal stem cells; AQP, Aquaporin; NF-κB, Nuclear factor-κB; TLR4, Toll-like receptor 4; SPAK, STE20/SPS1-related proline/alanine-rich kinase; VEGF, Vascular endothelial growth factor; PAR-1, Protease-activated receptor-1; p-Src, Phospho-Src; p-PAK1, Phospho-PAK1; BBB, Blood-brain barrier; VE-cadherin, Vascular endothelial-cadherin; CP, Choroidal plexus; Prx2, Peroxiredoxin 2; LPA, Lysophosphatidic acid; TRPV4, Transient receptor potential vanilloid 4; PPARγ, Peroxisome proliferator-activated receptor gamma; TGF-β1, Transforming growth factor-β1; ICA II, Icariside II; DFX, Deferoxamine; SCH79797, 3-N-cyclopropyl-7-[(4-propan-2-ylphenyl)methyl]pyrrolo[3,2-f]quinazoline-1,3-diamine;dihydrochloride; CTGF, Connective tissue growth factor; ICP, Intracranial pressure

References

[1] Ahn SY, Chang YS, Sung DK, Sung SI, Yoo HS, Im GH, *et al.* (2015). Optimal route for mesenchymal stem cells transplantation after severe intraventricular hemorrhage in newborn rats. PLoS ONE, 10.

[2] Lummis NC, Sánchez-Pavón P, Kennedy G, Frantz AJ, Kihara Y, Blaho VA, *et al.* (2019). LPA(1/3) overactivation induces neonatal posthemorrhagic hydrocephalus through ependymal loss and ciliary dysfunction. Sci Adv, 5:eaax2011.

[3] Guo J, Mi X, Zhan R, Li M, Wei L, Sun J (2018). Aquaporin 4 silencing aggravates hydrocephalus induced by injection of autologous blood in rats. Medical Science Monitor, 24:4204-4212.

[4] Zhang JB, Shi X, Chen Z, Geng JJ, Wang YL, Feng H, *et al.* (2018). Edaravone Reduces Iron-Mediated Hydrocephalus and Behavioral Disorder in Rat by Activating the Nrf2/HO-1 Pathway. JOURNAL OF STROKE & CEREBROVASCULAR DISEASES, 27:3511-3520.

[5] Meng H, Li F, Hu R, Yuan YK, Gong GQ, Hu SL, *et al.* (2015). Deferoxamine alleviates chronic hydrocephalus after intraventricular hemorrhage through iron chelation and Wnt1/Wnt3a inhibition. BRAIN RESEARCH, 1602:44-52.

[6] Karimy JK, Zhang J, Kurland DB, Theriault BC, Duran D, Stokum JA, *et al.* (2017). Inflammation-dependent cerebrospinal fluid hypersecretion by the choroid plexus epithelium in posthemorrhagic hydrocephalus. Nat Med, 23:997-1003.

[7] Shen D, Ye X, Li J, Hao X, Jin L, Jin Y, *et al.* (2022). Metformin Preserves VE–Cadherin in Choroid Plexus and Attenuates Hydrocephalus via VEGF/VEGFR2/p-Src in an Intraventricular Hemorrhage Rat Model. International Journal of Molecular Sciences, 23.

[8] Gao C, Du HJ, Hua Y, Keep RF, Strahle J, Xi GH (2014). Role of red blood cell lysis and iron in hydrocephalus after intraventricular hemorrhage. JOURNAL OF CEREBRAL BLOOD FLOW AND METABOLISM, 34:1070-1075.

[9] Gao F, Liu F, Chen Z, Hua Y, Keep RF, Xi G (2014). Hydrocephalus after intraventricular hemorrhage: the role of thrombin. J Cereb Blood Flow Metab, 34:489-494.
[10] Gao F, Zheng M, Hua Y, Keep RF, Xi G (2016). Acetazolamide Attenuates Thrombin-Induced Hydrocephalus. Acta Neurochir Suppl, 121:373-377.
[11] Hao XD, Le CS, Zhang HM, Shang DS, Tong LS, Gao F (2019). Thrombin disrupts vascular endothelial-cadherin and leads to hydrocephalus via protease-activated receptors-1 pathway. Cns Neuroscience & Therapeutics, 25:1142-1150.
[12] Peng K, Koduri S, Xia F, Gao F, Hua Y, Keep RF, *et al.* (2021). Impact of sex differences on thrombin-induced hydrocephalus and white matter injury: the role of neutrophils. Fluids and Barriers of the CNS, 18:38.
[13] Tan XX, Chen JY, Keep RF, Xi GH, Hua Y (2020). Prx2 (Peroxiredoxin 2) as a Cause of Hydrocephalus After Intraventricular Hemorrhage. Stroke, 51:1578-1586.
[14] Chen T, Tan X, Xia F, Hua Y, Keep RF, Xi G (2021). Hydrocephalus Induced by Intraventricular Peroxiredoxin-2: The Role of Macrophages in the Choroid Plexus. Biomolecules, 11.
[15] Toft-Bertelsen TL, Barbuskaite D, Heerfordt EK, Lolansen SD, Andreassen SN, Rostgaard N, *et al.* (2022). Lysophosphatidic acid as a CSF lipid in posthemorrhagic hydrocephalus that drives CSF accumulation via TRPV4-induced hyperactivation of NKCC1. Fluids Barriers CNS, 19:69.
[16] Bian CY, Wan YF, Koduri S, Hua Y, Keep RF, Xi GH (2023). Iron-Induced Hydrocephalus: the Role of Choroid Plexus Stromal Macrophages. TRANSLATIONAL STROKE RESEARCH, 14:238-249.
[17] Vinukonda G, Liao Y, Hu F, Ivanova L, Purohit D, Finkel DA, *et al.* (2019). Human Cord Blood-Derived Unrestricted Somatic Stem Cell Infusion Improves Neurobehavioral Outcome in a Rabbit Model of Intraventricular Hemorrhage. Stem Cells Translational Medicine, 8:1157-1169.
[18] Chua CO, Chahboune H, Braun A, Dummula K, Chua CE, Yu J, *et al.* (2009). Consequences of intraventricular hemorrhage in a rabbit pup model. Stroke, 40:3369-3377.
[19] Flores JJ, Klebe D, Rolland WB, Lekic T, Krafft PR, Zhang JH (2016). PPARγ-induced upregulation of CD36 enhances hematoma resolution and attenuates long-term neurological deficits after germinal matrix hemorrhage in neonatal rats. Neurobiology of Disease, 87:124-133.
[20] Lekic T, Klebe D, McBride DW, Manaenko A, Rolland WB, Flores JJ, *et al.* (2015). Protease-Activated Receptor 1 and 4 Signal Inhibition Reduces Preterm Neonatal Hemorrhagic Brain Injury. Stroke, 46:1710-1713.
[21] Guo J, Chen Q, Tang J, Zhang J, Tao Y, Li L, *et al.* (2015). Minocycline-induced attenuation of iron overload and brain injury after experimental germinal matrix hemorrhage. Brain Research, 1594:115-124.
[22] Lekic T, Manaenko A, Rolland W, Krafft PR, Peters R, Hartman RE, *et al.* (2012). Rodent neonatal germinal matrix hemorrhage mimics the human brain injury, neurological consequences, and post-hemorrhagic hydrocephalus. Exp Neurol, 236:69-78.
[23] Cui JJ, Wang D, Gao F, Li YR (2012). Effects of Atorvastatin on Pathological Changes in Brain Tissue and Plasma MMP-9 in Rats with Intracerebral Hemorrhage. Cell Biochemistry and Biophysics, 62:87-90.
[24] Chen Q, Tang J, Tan L, Guo J, Tao Y, Li L, *et al.* (2015). Intracerebral hematoma contributes to hydrocephalus after intraventricular hemorrhage via aggravating iron accumulation. Stroke, 46:2902-2908.
[25] Chen Q, Zhang J, Guo J, Tang J, Tao Y, Li L, *et al.* (2015). Chronic hydrocephalus and perihematomal tissue injury developed in a rat model of intracerebral hemorrhage with ventricular extension. Transl Stroke Res, 6:125-132.
[26] Jiang Y, Meng L, Yan J, Yue H, Zhu J, Liu Y (2021). Changes of arachnoid granulations after subarachnoid hemorrhage in cynomolgus monkeys. Journal of Integrative Neuroscience, 20:419-424.
[27] Nitta J, Tada T (1998). Ultramicroscopic Structures of the Leptomeninx of Mice with Communicating Hydrocephalus Induced by Human Recombinant Transforming Growth Factor-β1. Neurologia medico-chirurgica, 38:819-825.
[28] Tada T, Kanaji M, Shigeaki K (1994). Induction of communicating hydrocephalus in mice by intrathecal injection of human recombinant transforming growth factor-β1. Journal of Neuroimmunology, 50:153-158.
[29] Shibata Y, Kruskal JB, Palmer MR (2007). Imaging of cerebrospinal fluid space and movement of hydrocephalus mice using near infrared fluorescence. Neurological Sciences, 28:87-92.
[30] Chu SH, Feng DF, Ma YB, Zhang H, Zhu ZA, Li ZQ, *et al.* (2011). Expression of HGF and VEGF in the cerebral tissue of adult rats with chronic hydrocephalus after subarachnoid hemorrhage. Molecular Medicine Reports, 4:785-791.
[31] Dong C, Ming X, Ye Z, Wang P, Wang L, Li Z, *et al.* (2019). Icariside II Attenuates Chronic Hydrocephalus in an Experimental Subarachnoid Hemorrhage Rat Model. Journal of Pharmacy and Pharmaceutical Sciences, 21:318-325.
[32] Okubo S, Strahle J, Keep RF, Hua Y, Xi G (2013). Subarachnoid hemorrhage-induced hydrocephalus in rats. Stroke, 44:547-550.

© 2024. Liu X et al. Published online at http://www.aginganddisease.org/EN/10.14336/AD.2024.1434

[33] Wan Y, Hua Y, Garton HJL, Novakovic N, Keep RF, Xi G (2019). Activation of epiplexus macrophages in hydrocephalus caused by subarachnoid hemorrhage and thrombin. CNS Neuroscience and Therapeutics, 25:1134-1141.
[34] Shishido H, Zhang H, Okubo S, Hua Y, Keep RF, Xi G (2016). The Effect of Gender on Acute Hydrocephalus after Experimental Subarachnoid Hemorrhage. Acta Neurochir Suppl, 121:335-339.
[35] Goddeyne C, Nichols J, Wu C, Anderson T (2015). Repetitive mild traumatic brain injury induces ventriculomegaly and cortical thinning in juvenile rats. J Neurophysiol, 113:3268-3280.
[36] Zhao J, Chen Z, Xi G, Keep RF, Hua Y (2014). Deferoxamine attenuates acute hydrocephalus after traumatic brain injury in rats. Transl Stroke Res, 5:586-594.
[37] Makinde HM, Just TB, Cuda CM, Bertolino N, Procissi D, Schwulst SJ (2018). Monocyte depletion attenuates the development of posttraumatic hydrocephalus and preserves white matter integrity after traumatic brain injury. PLoS One, 13:e0202722.

© 2024. Liu X et al. Published online at http://www.aginganddisease.org/EN/10.14336/AD.2024.1434
